# A CRISPR activation screen identifies MUC-21 as critical for resistance to NK and T cell-mediated cytotoxicity

**DOI:** 10.1186/s13046-023-02840-9

**Published:** 2023-10-20

**Authors:** Dong-hee Lee, Hyejin Ahn, Hye-In Sim, Eunji Choi, Seunghyun Choi, Yunju Jo, Bohwan Yun, Hyun Kyu Song, Soo Jin Oh, Kaori Denda-Nagai, Chan-Sik Park, Tatsuro Irimura, Yoon Park, Hyung-seung Jin

**Affiliations:** 1 Department of Convergence Medicine, Asan Institute for Life Sciences, Asan Medical Center, University of Ulsan College of Medicine, Seoul, 05505 South Korea; 2 Chemical and Biological Integrative Research Center, Biomedical Research Institute, Korea Institute of Science and Technology (KIST), Seoul, 02792 South Korea; 3 Department of Life Sciences, Korea University, Seoul, 02481 South Korea; 4 Division of Glycobiologics, Graduate School of Medicine, Juntendo University, 2-1-1 Hongo, Bunkyo-ku, Tokyo, 113-8421 Japan; 5 Department of Pathology, Asan Medical Center, University of Ulsan College of Medicine, Seoul, 05505 South Korea

**Keywords:** Cancer immunotherapy, CRISPR library screening, MUC21, CAR

## Abstract

**Background:**

Immunotherapy has significantly advanced cancer treatments, but many patients do not respond to it, partly due to immunosuppressive mechanisms used by tumor cells. These cells employ immunosuppressive ligands to evade detection and elimination by the immune system. Therefore, the discovery and characterization of novel immunosuppressive ligands that facilitate immune evasion are crucial for developing more potent anti-cancer therapies.

**Methods:**

We conducted gain-of-function screens using a CRISPRa (CRISPR activation) library that covered the entire human transmembrane sub-genome to identify surface molecules capable of hindering NK-mediated cytotoxicity. The immunosuppressive role and mechanism of MUC21 were validated using NK and T cell mediated cytotoxicity assays. Bioinformatics tools were employed to assess the clinical implications of mucin-21 (MUC21) in cancer cell immunity.

**Results:**

Our genetic screens revealed that MUC21 expression on cancer cell surfaces inhibits both the cytotoxic activity of NK cells and antibody-dependent cellular cytotoxicity, but not affecting complement-dependent cytotoxicity. Additionally, MUC21 expression hinders T cell activation by impeding antigen recognition, thereby diminishing the effectiveness of the immune checkpoint inhibitor, anti-PD-L1. Moreover, MUC21 expression suppress the antitumor function of both CAR-T cells and CAR-NK cells. Mechanistically, MUC21 facilitates immune evasion by creating steric hindrance, preventing interactions between cancer and immune cells. Bioinformatics analysis revealed elevated *MUC21* expression in lung cancer, which correlated with reduced infiltration and activation of cytotoxic immune cells. Intriguingly, *MUC21* expression was higher in non-small cell lung cancer (NSCLC) tumors that were non-responsive to anti-PD-(L)1 treatment compared to responsive tumors.

**Conclusions:**

These findings indicate that surface MUC21 serves as a potent immunosuppressive ligand, shielding cancer cells from NK and CD8^+^T cell attacks. This suggests that inhibiting MUC21 could be a promising strategy to improve cancer immunotherapy.

**Supplementary Information:**

The online version contains supplementary material available at 10.1186/s13046-023-02840-9.

## Background

Immunotherapy has become a crucial treatment option for certain types of cancers. The success of immune-checkpoint inhibitors (ICI) and chimeric antigen receptor (CAR)-T cells in clinical trials has prompted a surge of research into other immunotherapeutic approaches [1, 2]. Despite the notable progress for patient outcomes from the use of these approaches to cancer treatment, a significant number of cancer patients continue to exhibit a lack of response to immunotherapeutics. This is partly attributed to immunosuppressive mechanisms used by these cancer cells [3, 4]. Hence, it has become vital to identify these immunosuppressive mechanisms and thereby develop new strategies for overcoming immunotherapy resistance in cancer patients.

The CD8^+^ cytotoxic T cells of the adaptive immune system play a pivotal role in anti-tumor immunity. These cells can eliminate cancer cells by recognizing peptide antigens presented by major histocompatibility complex (MHC) class I on their surfaces. Several reports have now demonstrated that mutations in this antigen presentation machinery, particularly the loss of MHC or β-2 microglobulin (β2M), are involved in the evasion by cancer cells of T cell-mediated immune surveillance [5, 6]. Natural killer (NK) cells, which are part of the innate immune system, serve a complementary function to CD8^+^T cells through the targeting of cancer cells that lose MHC-I and can thus avoid CD8^+^T cell responses [7]. The function of NK cells is tightly regulated by germline-encoded activating and inhibiting receptors that interact with various ligands expressed on the surface of the target cells. The main classes of NK receptors include natural cytotoxicity receptors (NCR), killer-cell lectin-like receptors (KLR), and killer-cell immunoglobulin-like receptors (KIR) [8]. The fragment crystallizable (Fc) receptor (FcR) on NK cells (CD16 encoded by *FCRG3A*) recognizes the Fc portion of the cell surface bound IgG antibody, which activates NK cells for antibody-dependent cell-mediated cytotoxicity (ADCC). Therapeutic monoclonal antibodies are primarily designed to trigger NK cell-mediated ADCC against cancer cells. Complement-dependent cytotoxicity (CDC) is also an important mechanism of action for monoclonal antibodies, whereby they activate the complement system, leading to the specific lysis of target cells.

Cancer cells express various cell surface proteins that interact with inhibitory receptors on immune cells, thereby protecting themselves from elimination by the immune system. The comprehension of receptor-ligand interactions during immune responses has resulted in the development of blocking antibodies, such as anti-programmed cell death receptor ligand 1 (PD-L1) and anti-PD-1 antibodies, which obstruct inhibitory signaling in immune cells. This advancement has significantly enhanced the prognosis of cancer patients [9]. However, given that numerous activating and inhibitory cell membrane receptors are expressed heterogeneously in cancer and immune cells, further characterization of these receptors and ligands is imperative to facilitate the development of novel and efficacious anti-cancer treatments.

CRISPR-Cas9 screens have recently been utilized to identify as yet unknown mechanisms that play crucial roles in determining the sensitivity or resistance of tumors to immune cells [10, 11]. Most of these screens rely on the inactivation of coding genes, but such CRISPR loss-of-function screens may fail to identify genes with low basal expression or those that are lethal when completely lost. However, these limitations can be addressed by employing gain-of-function screens using a CRISPR activation (CRISPRa) library. In CRISPRa, a catalytically inactive Cas9 protein (dCas9) is fused with a transcriptional activator, enabling a transient increase in gene expression at specific genomic sites by modulating the endogenous promoter [12–14].

In this present study, we performed gain of function screens with a CRISPRa library covering the entire human transmembrane sub-genome, with the aim of identifying surface molecules that inhibit NK-mediated cytotoxicity. We identified MUC21, which protects cancer cells from NK and CD8^+^T cell mediated killing. We found that MUC21 plays a key role in immune system evasion by creating steric hindrance that prevents the binding of cancer cells by immune cells. Moreover, our bioinformatics analysis revealed an increased expression of *MUC21* in lung cancer, which correlated with decreased immune cell infiltration. Intriguingly, high levels of *MUC21* expression in certain non-responder patients were indicative of their unresponsiveness to anti-PD-(L)1 immune checkpoint inhibitors, emphasizing the need to target the inhibition of MUC21 for more effective immunotherapies.

## Methods

### Cell lines and culture

The K562, Raji, NCI-H441, NCI-H1563, NCI-H1299, NCI-H2347, A549, p815 and NK-92 cell lines were obtained from the American Type Culture Collection (ATCC), while the 293FT and Expi293F™ cells were purchased from Thermo Fisher Scientific (#R70007). All cells were maintained according to the provider’s recommendations and were cultured for approximately up to 20 passages. NK-92 cells were cultured in Minimum Essential Medium alpha supplemented with 15% FBS, 15% horse serum, and additional supplements, including myo-inositol (0.2 mM), 2-mercaptoethanol (0.1 mM), folic acid (0.02 mM), and interleukin (IL)-2.

### Lentiviral production and transduction

K562 cells expressing VP64-dCas9-VP64 and MS2-p65-HSF1 (referred to as K562-V2M) were generated by transfecting them with the pPB-R1R2_EF1aVP64dCas9VP64_T2A_MS2p65HSF1-IRESbsdpA plasmid (Addgene plasmid #113341) along with pCMV(CAT)T7-SB100 (Addgene plasmid #34879) using the Neon electroporation system from Thermo Fisher Scientific. After a month of blasticidin selection, a single clone demonstrating the highest potential for transcription activation was selected for further study using the limiting dilution method and flow cytometry analysis of the PD-1 protein after infecting with two different sgRNAs targeting the *PD-1* promoter regions. The Wright Human Membrane Protein Activation Library (Addgene plasmid #113345), a gift from Dr. Gavin Wright, contains 58,570 sgRNAs targeting the promoter regions of 6,213 membrane proteins and 500 non-targeting sgRNAs [12]. This library was amplified and sequenced using an Illumina sequencer to confirm the distribution of sgRNAs in accordance with the manufacturer’s protocol. To generate lentivirus, 5 × 10^6^ 293FT cells were seeded into a 10 cm dish on day 0 and transfected with 7 µg of the library plasmid, 5 µg of psPAX2 (Addgene plasmid #12260), and 2 µg of pMD2.G (Addgene plasmid #12259) using 21.7 µL of Lipofectamine 3000 and 28 µL of P3000 reagent diluted in Opti-MEM media from Thermo Fisher Scientific. At 14 h post-transfection, the culture medium was changed to fresh DMEM supplemented with 10% FBS. The virus-containing medium was collected 2 days later, filtered through a 0.45 μm syringe, and stored at -80 °C until further use. For the pooled screening, 1 × 10^8^ K562-V2M cells were spin-infected with the lentivirus at a multiplicity of infection (MOI) of 0.3, which corresponds to 500× library coverage. The following day, infected cells were replenished with fresh complete medium containing 4 µg/mL puromycin (MilliporeSigma) every 2 days to remove uninfected cells. At day 2, the infection rate was confirmed to be approximately 30% by analyzing BFP-positive cells using flow cytometry. Five days after post-infection, cells were washed with fresh complete medium to remove dead cell debris, and the proportion of BFP-positive cells was confirmed to be approximately 95%. A total of 3 × 10^7^ cells, equivalent to a 500× library coverage, were used for the pooled screening and next generation sequencing (NGS) analysis as a negative control, respectively.

### CRISPRa screen using NK-92 cells

NK-resistant K562 cells were screened by co-culturing the sgRNA-transduced pool of K562-V2M cells with NK-92 cells. In brief, a 2 × 10^6^ sgRNA-transduced pool of K562-V2M cells was seeded into a 10 cm dish with 1 × 10^6^ NK-92 cells and recombinant human IL-2. A total of 30 dishes were incubated in a humidified CO^2^ incubator for 2 days. The following day, the mixed cells were washed twice with complete growth medium to remove recombinant human IL-2. Surviving K562 cells were enriched in complete growth media without recombinant human IL-2 until the cell number reached 3 × 10^7^. Genomic DNA was extracted from 3 × 10^7^ enriched cells using the DNeasy Blood & Tissue Kit (Qiagen). NGS libraries were generated by amplifying the genomic DNA with NEBNext High-Fidelity 2X PCR Master Mix (New England BioLabs). PCR products were purified using AMPure XP beads (Beckman Coulter) and quantified using a Bioanalyzer (Agilent). An Illumina sequencer platform generated over 15 million total reads per sample, and the resulting sequencing FASTQ data were analyzed using MAGeCK software [4]. Candidate genes were identified based on their positive MAGeCK score and false discovery rate (FDR), comparing the negative control to the second enriched cells.

### Plasmids

The full-length human *MUC21* gene (SinoBiological, HG24673-UT) was PCR-amplified, cloned with a Myc epitope at the N-terminus, and inserted into pSB-tet-RB (Addgene plasmid #60506). To generate recombinant MUC21-mIgG2a (rMUC21-mFc), the extracellular domain of human *MUC21* gene (amino acids 25–479) was PCR-amplified and cloned into the pFUSE-mIgG2a-Fc vector (Invivogen). Additionally, the human CD16a/Fc gamma RIIIa cDNA (SinoBiological, HG10389-G) was PCR-amplified and inserted into the pSBbi-BP vector (Addgene plasmid #60512). To generate a plasmid encoding the A02:01/NY-ESO-1 single-chain trimer [15], a single-stranded oligonucleotide encoding a NY-ESO-1 peptide (157–165) (TCCCTGCTGATGTGGATCACCCAGGTG) was inserted into the BsmBI-digested pCCLc-MND-A0201-SABR-Backbone vector (Addgene plasmid #119050). The resulting construct was then amplified using primers that encompass the signal sequence, NY-ESO-1 peptide, Beta-2-microglobulin, HLA-A*02:01, and transmembrane domain. Finally, the PCR product was inserted into the pSBbi-BP vector (Addgene plasmid #6051). The CD19 CAR contained the scFv against human CD19 (clone FMC-63), linked to 4-1BB and CD3ζ intracellular domains via a CD8 transmembrane domain and CD8-hinge. The gene fragment encoding CD19 CAR was synthesized and subsequently cloned into the pHR-PGK vector (Addgene plasmid #79125). Guide RNA sequences for activating the transcription of the target gene were acquired from the Wright Human Membrane Protein Activation Library. Oligonucleotides, designed to target the promoter regions of *PDCD1* (#2: GGGTGAGGAGGGGGTAGGAC, #3: GGGGAGAGAGAGACAGAGAC) were inserted into the pKVL2-U6gRNA_SAM(BbsI)-PGKpuroBFP-W plasmid (Addgene plasmid #112925) to enable lentiviral expression of the gRNAs. RNA interference-mediated knockdown of *MUC21* was achieved using pHR lentiviral vectors containing H1 promoter. The specific sequences of the shRNA hairpins used were as follows: *MUC21* shRNA: 5’-GCAACAAATTCCAATGAGActtcctgtcagaTCTCATTGGAATTTGTTGC-3’, Scramble: 5’-GACGAGCGGCACGTGCACActtcctgtcagaTGTGCACGTGCCGCTCGTC-3’. All of these constructs were verified by Sanger sequencing.

### Stable cell line generation

To conduct the killing assay, target cells, including the K562, Raji, NCI-H441, p815 and A549, were transduced with a plasmid containing the firefly luciferase gene using the Sleeping Beauty transposon system [16]. For the generation of doxycycline (Dox)-inducible MUC21-expressing K562, Raji, p815 and A549 cell lines, the pSBtet-RB-MUC21 plasmid was transfected into the cells along with pCMV(CAT)T7-SB100. The MUC21-expressing cells were then sorted using anti-MUC21 antibodies (heM21C). In the case of NCI-H441 cells, *MUC21* was knockdown by the lentivirus-mediated shRNA approach, and the resulting *MUC21* knockdown NCI-H441 cells were selected via FACS sorting.

### FACS analysis

Human single cells were stained with fluorochrome-conjugated antibodies after pre-blocking with human TruStain FcX™ (BioLegend) in FACS buffer (PBS with 1% BSA and 0.1% sodium azide) for 20 min at 4 °C. For intracellular staining, the cells were fixed and permeabilized using a Cytofix/Cytoperm Kit (BD Biosciences).The following fluorochrome-conjugated antibodies were purchased from BioLegend: anti-CD3 (HIT3a), anti-CD8 (SK1), anti-CD16 (3G8), anti-CD19 (HIB19), anti-CD20 (rituximab), anti-CD25 (BC96), anti-CD56 (5.1H11), anti-CD69 (FN50), anti-CD107a (H4A3), anti-CD226(11A8), anti-PD-1 (EH12.2.H7), anti-TIGIT (A15153G), anti-SLAMF7 (162.1), anti-Granzyme B(GB11), anti-IFN-γ (4 S.B3), anti-TNF-α (W19063E) and anti-PD-L1 (29E.2A3).

### ADCC assay

For the ADCC assay, Raji-tet-MUC21 cells were treated with rituximab (BioXcell), NCI-H441 cells (shCTL and sh*MUC21*), and A549-tet-MUC21 were treated with cetuximab (BioXcell) at 37 °C for 30 min. Subsequently, NK-92-CD16 cells were added and co-cultured with the treated cells at 37 °C with 5% CO2 for 4–5 h. The killing activity was measured by assessing the luciferase activity of viable cells. Luminescent signals emitted from the cell lysates were quantified using the Luciferase Assay System (Enzynomics) according to the manufacturer’s instructions.

### CDC assay

Raji-tet-MUC21 cells were employed as the target cells in the CDC assays, while human complement (MilliporeSigma, S1764) was utilized as the complement source. The target cells were combined with different concentrations of rituximab, and subsequently, 10% human complement was added. After incubating the mixture for 4 h, cell viability was measured using a luciferase assay.

### Human T cell assay

Cryopreserved human peripheral blood mononuclear cells (PBMCs) were purchased from ImmunoSpot (Cleveland, OH). Human CD3^+^T cells were separated from PBMCs using an EasySep Human T cell Isolation Kit (STEMCELL Technologies). For experiments where T cell proliferation was measured, purified CD8^+^T cells were labeled with CellTrace Violet (CTV; Invitrogen) prior to cell culture. The proliferation of CTV-labeled CD8^+^T cells was evaluated by quantification of CTV dilution. The CD8^+^T cells were stimulated with 293FT cells expressing MUC21 in the presence of plate-coated anti-CD3 antibody (Clone: OKT3, BioLegend) or p815-OKT3-tet-MUC21 as artificial antigen presenting cell. IFN-γ secretion in culture medium was analyzed by ELISA (BioLegend).

### 1G4 TCR-T cell assay

A lentivirus encoding a 1G4 T cell receptor (TCR) that recognizes the HLA-A∗0201 restricted NY-ESO-1 peptide (NY-ESO-1:157–165) was transduced into human primary CD8^+^T cells. The expression of 1G4 TCR was then assessed by flow cytometric analysis using an anti-human Vβ13.1 TCR chain antibody (BioLegend) [16]. 1G4 TCR-engineered CD8^+^T cells were co-cultured with Raji cells stably expressing A*02:01/NY157–165 single-chain trimers, human PD-L1 and Dox-inducible MUC21 (Raji-A2-ESO-1-PD-L1-MUC21) for three days in the presence of anti-PD-1 (pembrolizumab biosimilar, BioXcell), anti-PD-L1 (atezolizumab biosimilar, BioXcell) or anti-4-1BB (urelumab biosimilar, ichorbio) antibodies.

### Generation of CD19 targeting CAR-T and CAR-NK cells

To produce lentivirus encoding the CD19 CAR gene, 293FT cells were transfected with pHR**-**PGK-CD19 CAR along with the packaging plasmids psPAX2 (Addgene plasmid #122160) and pVSVg (Addgene plasmid #8484) using Lipofectamine 3000 (Invitrogen). After 72 h of transfection, lentiviral supernatants were collected and subsequently filtered through a 0.45 μm PES membrane. To generate human primary anti-CD19 CAR-T cells, human CD3^+^T cells were isolated from human PBMCs using negative selection (STEMCELL Technologies) and then activated with Dynabeads™ Human T-Activator CD3/CD28 (Invitrogen) at cell-to-bead ratio of 1:1 and IL-2 (100 ng/ml; Peprotech) in complete RPMI 1640 medium. Three days after activation, activated CD3^+^T cells were transduced with lentiviral supernatants in the presence of polybrene (8 µg/ml). After 48 h, the lentiviral supernatants containing polybrene and Dynabeads were removed and the transduced CD19 CAR-T cells were maintained in complete RPMI 1640 medium with 100 ng/ml IL-2. The transduction efficiency of CD19 CAR-T cells was measured by flow cytometric analysis using biotinylated recombinant human CD19-His protein (Sino Biological) with PE-conjugated streptavidin (BioLegend). To generate anti-CD19 CAR-NK92 cells, NK-92 cells were transduced with lentiviral supernatants in the presence of polybrene (8 µg/ml). After 48 h, the lentiviral supernatants containing polybrene were removed and the transduction efficiency of anti-CD19 CAR-NK-92 cells was measured as described above.

### Ex vivo expansion of NK cells

Human PBMCs (1.5 × 10^6^) were co-incubated with 100 Gy-irradiated K562 cells (1 × 10^6^) in RPMI1640 medium supplemented with 100 U/mL hIL-2. The medium was refreshed every two days with fresh medium containing recombinant IL-2. After one week, residual T cells were depleted using a CD3 positive selection kit (BioLegend). Purified NK cells were then incubated in medium with 5 ng/mL hIL-15 for an additional two weeks, with medium exchange every two days. Flow cytometry analysis confirmed the purity of the expanded cell populations.

### Cell conjugation assay

Prior to conducting the cell conjugation assay, K562-tet-MUC21 cells were treated with Dox (MilliporeSigma) for 24 h. NK-92 cells were labeled with CTV. After this labeling, the NK-92 cells and K562-tet-MUC21 cells were combined in a 1:1 ratio and incubated for 15 min at 37 °C. The formation of conjugates was promptly analyzed using flow cytometry [17]. The conjugation between Raji-tet-MUC21 and CD19 CAR-T cells was measured using the same approach.

### Expression and purification of recombinant proteins

Expi293F cells were cultured in Expi293 expression medium (A1435101; Thermo Fisher Scientific) in a shaking incubator at 37℃ with 8% CO_2_. Transfection of Expi293F cells was conducted using the Expi293™ Expression System Kit (A146315; Thermo Fisher Scientific) following the manufacturer’s protocol. The protein was purified from the cell culture supernatant using recombinant Protein A affinity chromatography (HiTram MabSelect SuRe, 28-4082-55; GE Healthcare).

### 3D culture

The culture involves a 2:1 mixture of VitroGel® Hydrogel Matrix (Well Bioscience Inc.) and culture media at room temperature, with a cell density of 2 × 10^6^ cells/ml. The media supplement concentration is tripled when mixed with the Hydrogel Matrix. NCI-H441 cells, resuspended in DMEM at a density of 4 × 10^6^ cells/mL, are mixed with the Hydrogel Matrix in a 2:1 ratio. These cells are then seeded onto a 24-well plate at a density of 4 × 10^5^ cells per 300 µl of the mixture and incubated at room temperature for 30 min without shaking. For FACS analysis, cells are harvested using the VitroGel® Cell Recovery Solution (Well Bioscience Inc.).

### Western blot analysis

NK-92 cells were serum-starved for 2 h without IL-2 and then mixed with paraformaldehyde-fixed K562 cells at a 1:0.5 (T:E) ratio and stimulated for 10 min. Cell lysates were prepared using SDS sample buffer (IBS-BS002; Intron), separated by SDS-PAGE, and subjected to western blot analysis using specific antibodies, including phospho-AKT (#4060) and β-actin (#8457) from Cell Signaling Technology.

### *MUC21* gene expression analysis

*MUC21* gene expression analyses of tumor and normal samples in The Cancer Genome Atlas (TCGA) and from Genotype-Tissue Expression (GTEx) were performed on the GEPIA2 web portal (http://gepia2.cancer-pku.cn/) and UCSC Xena browser (https://xenabrowser.net/). The gene expression data of 1372 Cancer Cell Line Encyclopedia (CCLE) cell lines were obtained from the Dependency Map (DepMap) Public 21Q1 dataset on the DepMap web portal (https://depmap.org/portal/). Normal tissue gene expression data were obtained from the GTEx portal (https://gtexportal.org/). *MUC21* promoter methylation profiles in TCGA samples were analyzed and visualized on MEXPRESS (https://mexpress.be/) [18].

### Immune cell infiltration analysis

The correlation between *MUC21* gene expression and immune cell infiltration of lung adenocarcinoma and lung squamous cell carcinoma were estimated with multiple deconvolution methods from TCGA RNA sequencing data on the TIMER2.0 web portal (http://timer.cistrome.org/). TIMER, CIBERSORT, xCell, MCP-counter, quanTIseq, and EPIC algorithms were used for these estimations [7]. The results were visualized using heatmaps with R software (version 4.1.1).

### Survival analysis

Survival analysis was performed using the UCSC Xena browser to compare the survival outcomes between the *MUC21*-high and *MUC21*-low groups in lung cancer patients from TCGA. Briefly, TCGA patient samples were subgrouped into two cohorts based on 25% cutoff expression values. The overall survival (OS) and disease-free survival (DFS) of these two cohorts were visualized using Kaplan–Meier curves, along with hazard ratios (HRs) obtained from the Cox proportional hazards model and log-rank *P*-values.

### Patient data analysis

Gene expression and clinical data of NSCLC patients who underwent anti-PD-1 or anti-PD-L1 antibody therapy were downloaded from the Gene Expression Omnibus (https://www.ncbi.nlm.nih.gov/geo/): GSE126044 and GSE135222. The *MUC21* gene expression profile of each patient was plotted against their clinical response to anti-PD-1 or anti-PD-L1 therapy using the ggplot2 R package.

### Statistical analysis

Statistical analysis was conducted using GraphPad Prism (version 9.2.0) software and R programming language (version 4.1.1, https://www.r-project.org/). Significance was determined using the two-tailed paired or unpaired Student *t*- test, or one-way or two-way ANOVA with multiple comparisons, and the log-rank test for the survival analysis. *P* values < 0.05 were considered statistically significant (*< 0.05, **< 0.01, ***< 0.001, and ****< 0.0001).

## Results

### A surfaceome CRISPR activation screen for candidate genes that confer resistance to NK cell cytotoxicity

**Fig. 1:**
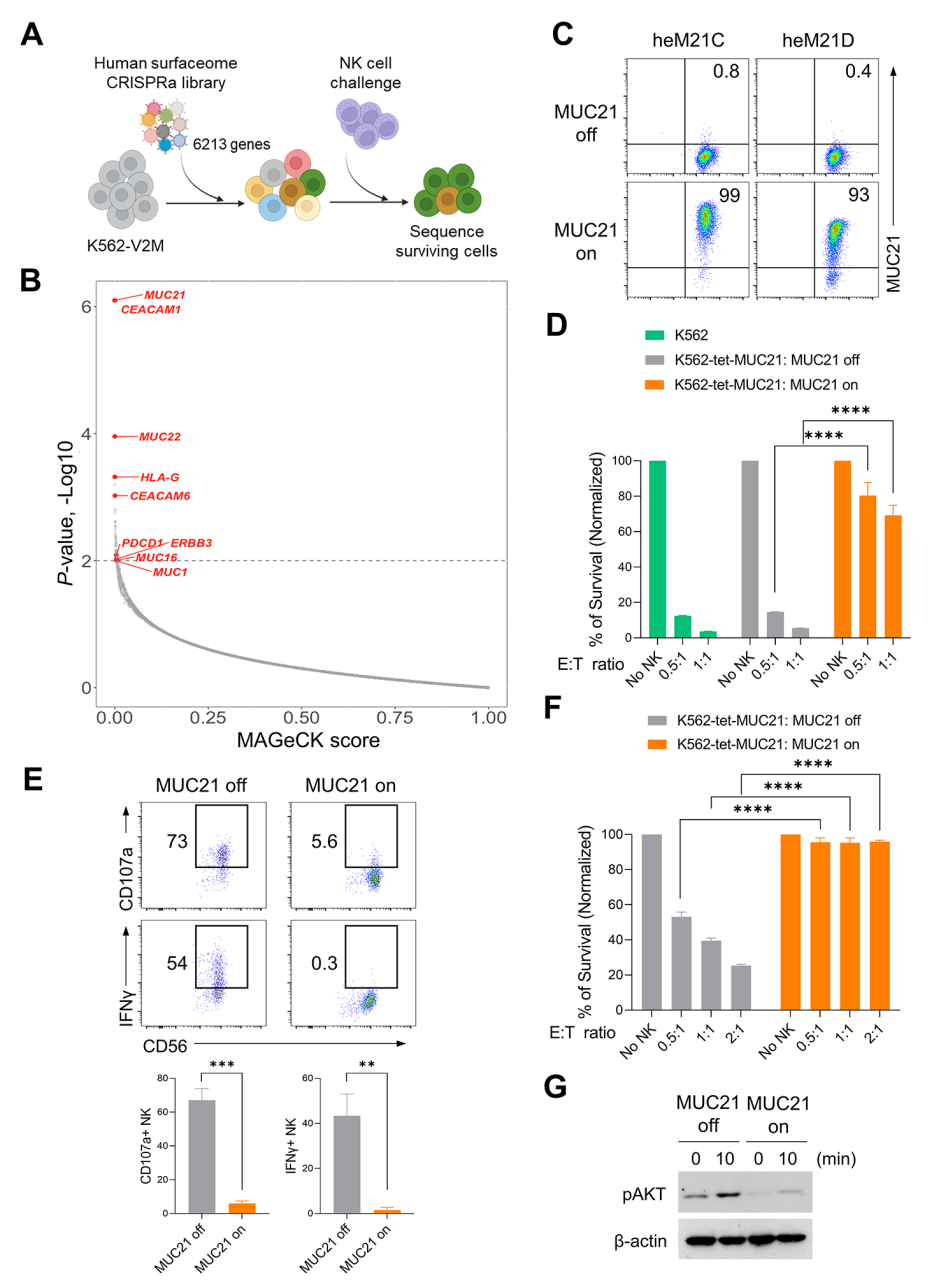
Identification of candidate genes conferring resistance to NK cell cytotoxicity through a surfaceome CRISPR activation screen. (**A**) Overview of the surfaceome-focused CRISPRa screen used in this study. A lentiviral library comprising 58,071 sgRNAs targeting the promoter regions of 6213 cell surface protein genes and 500 control sgRNAs was employed. K562-V2M cells were transduced with the sgRNA library and exposed to NK-92 cells for two days. Genomic DNA was extracted from the cells that survived, and gene abundance was determined using next-generation sequencing. (**B**) MAGeCK Analysis of the enrichment of sgRNA sequences in the surviving K562-V2M cells. X-Axis: MAGeCK Gene Score; Y-Axis: inverse log *P* value. (**C**) Representative FACS analysis of surface MUC21 expression in K562-tet-MUC21 cells after 24 h of culture with or without Dox (1 µg/mL). Two different clones of antibodies (clones heM21C and heM21D) targeting human MUC21 were used. (**D**-**E**) K562-tet-MUC21 cells were cultured in the presence of Dox (1 µg/mL) for 24 h, and co-incubated with NK-92 cells for 6 h. (**D**) NK cell-mediated killing activity was evaluated by measuring the luciferase activity of the surviving K562 cells. Parental K562 cells were used as a positive control. Ratio of effector to target cells (E:T). (**E**) Representative FACS analysis (above panel) of the surface CD107a and intracellular IFN-γ expression in NK-92 cells. A summary graph (below panel) showing the percentage of CD107a and IFN-γ expressing NK-92 cells in two independent experiments. (**F**) Dox-treated K562-tet-MUC21 cells were exposed to primary NK cells at a 1:1 E:T ratio for six hours. NK cell killing was evaluated by measuring luciferase activity in surviving K562-tet-MUC21 cells. (G) Immunoblotting for phospho-AKT expression in lysates from NK-92 cells after stimulation with paraformaldehyde-fixed K562-tet-MUC21 cells for 0 and 10 min. β-actin was used as a loading control. Statistical significance was determined by two-tailed unpaired *t*-tests; **P < 0.01, ***P < 0.001, ****P < 0.001

We conducted a surfaceome-focused CRISPRa screen of K562 cells to identify tumor ligands that modulate NK cell-mediated killing. K562 cells are a NK-sensitive chronic myelogenous leukemia cell line that is deficient in MHC class I expression. For this screening process, we generated a clonal isolate of K562 cells expressing high levels of the double VP64-dCas9-VP64 activator construct, comprising VP64 transcriptional activators fused with a deactivated Cas9 (dCas9) and MS2-p65-HSF1 fusion proteins [12]. Infection of the activator stable cell line (K562-V2M) with lentiviruses encoding single guide RNAs (sgRNA) corresponding to human PD-1 resulted in the upregulation of surface PD-1 expression, thus validating the suitability of K562-V2M cells for the CRISPRa screen (Suppl. Figure S1A). The plasmid library was sequenced to confirm complete representation of total sgRNA complexity (Suppl. Figure S1B). Subsequently, K562-V2M cells were infected with a surfaceome CRISPRa library targeting the promoter regions of 6213 genes, all encoding known cell surface proteins, along with 500 non-targeting controls. At seven days post-infection, the cells harboring the CRISPRa library or control cells were co-cultured with NK-92 cells at an E:T ratio of 1:2 for 48 h (Fig. 1A). Deep sequencing and MAGeCK analysis were used to analyze the distribution of the sgRNA library in the surviving cells (Fig. 1B). The screen identified *MUC21* as the top-ranked NK cell evasion mechanism. Additionally, we observed significant enrichment of sgRNAs targeting carcinoembryonic antigen-related cell adhesion molecule 1 (*CEACAM1*) and nonclassical histocompatibility antigen *HLA-G*, known to inhibit NK cell activity [19, 20]. Besides *MUC21*, sgRNAs targeting various mucin family genes, such as *MUC22*, *MUC16* and *MUC1*, were further found to be enriched after NK cell challenge [21, 22]. MUC21 is known as a membrane-bound mucin that forms mucous barriers on epithelial cells that play an essential role in protecting the surfaces of body tracts [23]. Given the limited understanding of how mucin molecules expressed in tumor cells modulate NK cell cytotoxicity, we focused on MUC21 for further analysis. To validate the CRISPR screen, we generated K562 stable cells with Dox-inducible expression of MUC21 (K562-tet-MUC21). FACS analysis confirmed the Dox-induced upregulation of MUC21 using heM21D and heM21C antibodies (Fig. 1C). The results indicated that K562-tet-MUC21 expressed glycosylated MUC21, as the heM21C antibody could not bind to the unmodified core polypeptide of MUC21 [24]. To investigate the influence of MUC21 on NK-mediated cytotoxicity, Dox-treated and non-treated K562-tet-MUC21 cells were subjected to killing by NK-92 cells. The expression of MUC21 in K562 cells provided significant protection against NK cell-mediated killing (Fig. 1D). We also observed a substantial decrease in surface CD107a and intracellular IFN-γ expression in NK-92 cells when they were challenged with Dox-treated K562-tet-MUC21 cells (Fig. 1E). We confirmed that MUC21 overexpression in K562 cells conferred resistance to primary NK cells (Figs. S2 and 1 F). Considering the critical role of the phosphoinositide-3-kinase (PI3K)/AKT pathway in the effector functions of NK cells [25], we next investigated whether the presence of MUC21 on target cells could affect the activation of this pathway in NK cells. We stimulated NK-92 cells with fixed K562 cells or K562 cells expressing MUC21 and evaluated the levels of activated AKT. Notably, the exposure of NK-92 cells to K562-MUC21 cells resulted in a significant decrease in AKT activation compared to exposure to the K562 control cells (Fig. 1G). These findings suggested that the upregulation of MUC21 on tumor cells strongly inhibits NK cell effector functions.

### Membrane-bound MUC21 on cancer cells inhibits ADCC but not CDC

**Fig. 2:**
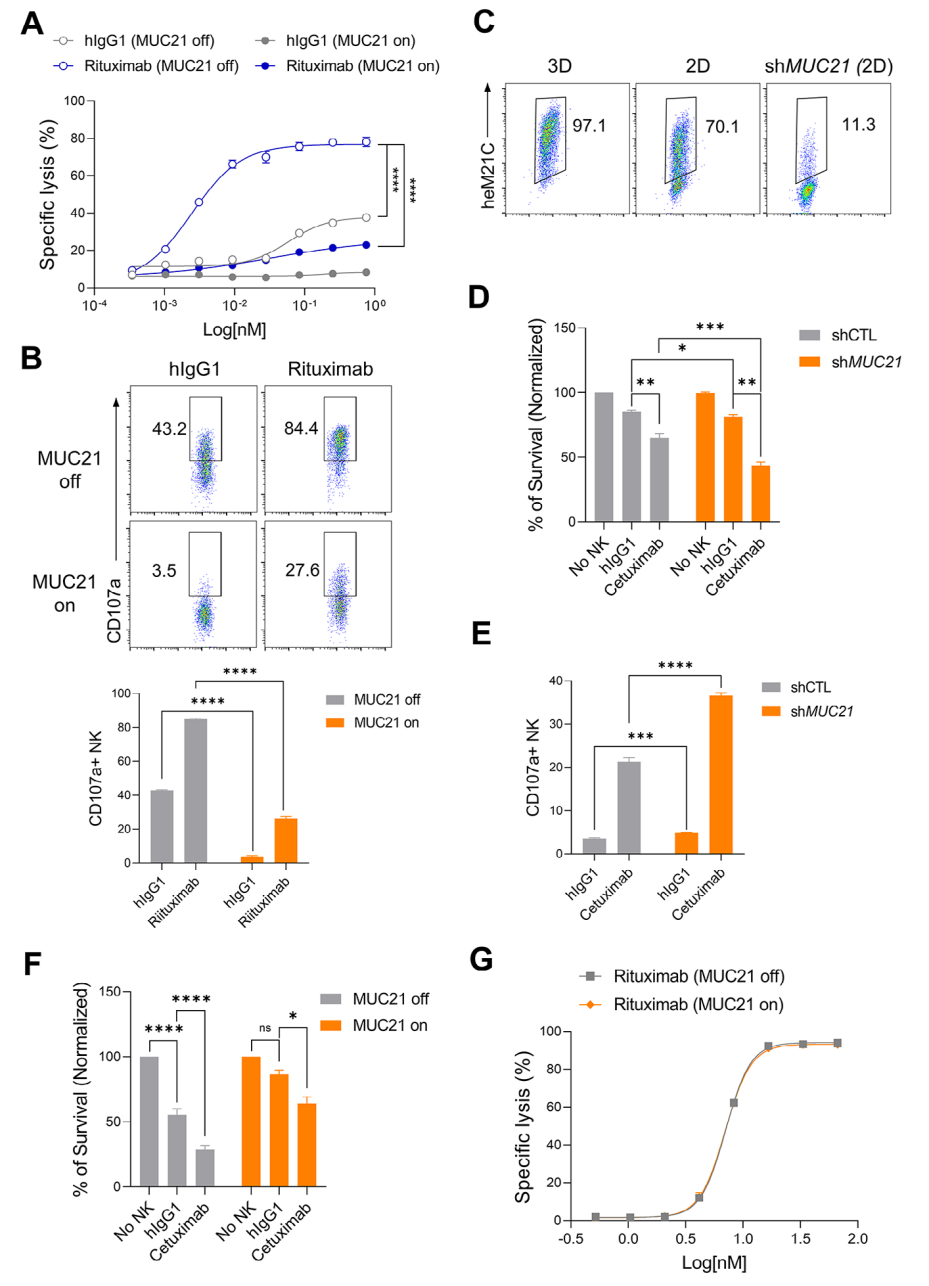
Surface MUC21 on cancer cells suppresses ADCC activity but has no impact on CDC. (**A-B**) Raji-tet-MUC21 cells were cultured with or without Dox (1 µg/mL) for 24 h. (**A**) Raji-tet-MUC21 cells were co-incubated with NK-92-CD16 cells at an E:T ratio of 0.5:1 for 4 h in the presence of varying concentrations of human IgG1 or rituximab. NK-92-CD16 cytotoxicity against Raji cells was measured by the luciferase activity of the surviving Raji cells. (**B**) Representative FACS analysis (above panel) of the surface CD107a expression of NK-92-CD16 cells cultured with Raji-tet-MUC21 cells in the presence of 10 µg/ml of hIgG1 or rituximab. A summary graph (below panel) showing the percentage of CD107a expressing NK-92 cells. (**C**) FACS analysis of surface MUC21 expression in wild-type NCI-H441 cultured in both 2D and 3D conditions, as well as *MUC21* knockdown NCI-H441 cells (sh*MUC21*) cultured in 2D. (**D**-**E**) The NCI-H441 cells, which were stably expressing scramble shRNA (shCTL) or shRNA targeting *MUC21* (sh*MUC21*), were cultured in 2D condition with NK-92-CD16 cells at an E:T ratio of 0.5:1 for 4 h. The co-culture was conducted in the presence of human IgG1 (0.1 µg/ml) or cetuximab (0.1 µg/ml). (**D**) NK-92-CD16 cytotoxicity against NCI-H441 cells was measured by luciferase activity in surviving NCI-H441 cells. (**E**) A summary graph of surface CD107a expression of NK-92-CD16 cells. (**F**) A549-tet-MUC21 cells were cultured with or without Dox (1 µg/ml) for 24 h and then co-incubated with NK-92-CD16 cells at an E:T ratio of 0.5:1 for 4 h in the presence of either human IgG1 (0.1 µg/ml) or cetuximab (0.1 µg/ml). (**G**) Raji-tet-MUC21 cells were cultured with or without Dox (1 µg/ml) for 24 h and incubated with indicated concentration of rituximab for 15 min followed by addition of 10% human complement. Cell viability was then measured by luciferase activity. Statistical significance was determined by a two-way ANOVA with Holm-Sidak comparisons in (**A**) or one-way ANOVA with Holm–Sidak multiple comparisons in (**B**), (**D**) and (**E**) or multiple *t* tests with correction for multiple comparisons using the Holm–Sidak method in (**F**). *P < 0.01, **P < 0.01, ***P < 0.001, ****P < 0.001; and ns, not significant

**Fig. 3:**
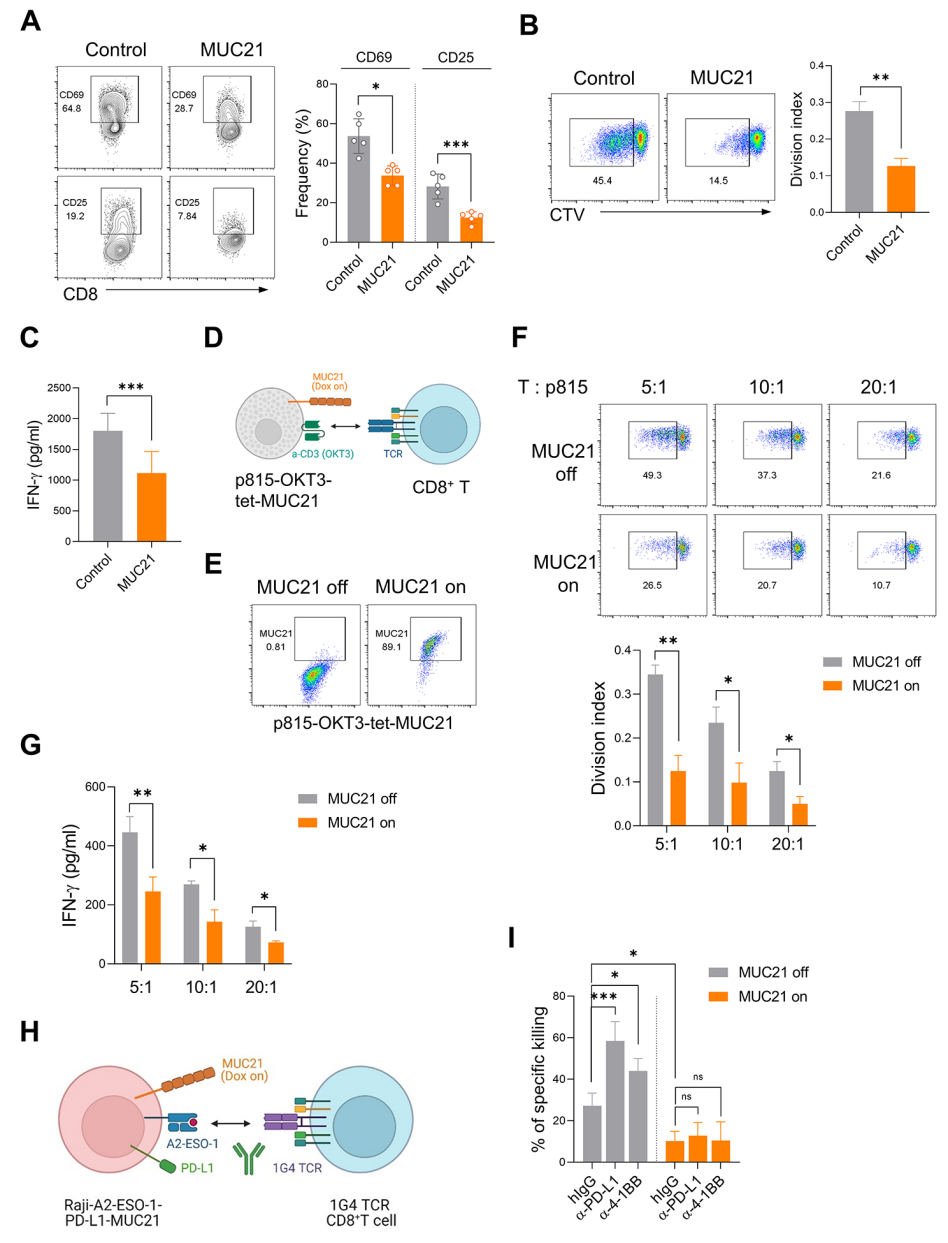
MUC21 attenuates T cell activation by hindering their antigen recognition. (**A**-**C**) CD8^+^T cells isolated from human PBMCs were co-cultured with 293FT cells that were transfected with mock (control) or MUC21 expressing plasmids for five days. The co-culture was performed in the presence of plate-coated anti-CD3 antibody (3 µg/ml). (**A**) Representative FACS plots (left panel) and a summary graph (right panel) showing the percentages of CD69^+^ or CD25^+^ CD8^+^T cells. Each dot represents an individual human sample. (**B**) Representative FACS plots showing proliferating CTV_low_ CD8^+^T cells (left panel) and a summary graph showing the division index of CTV_low_ CD8^+^T cells (right panel). (**C**) ELISA of IFN-γ secretion by CD8^+^T cells. (**D**-**G**) CD8^+^T cells were isolated from human PBMCs and co-cultured with p815 cells expressing membrane bound anti-CD3 scFv and Dox inducible MUC21 (p815-OKT3-tet-MUC21) at various E:T ratios for 3 days, in the presence or absence of Dox (1 µg/ml). (**D**) Schematic illustration of the p815-OKT3-tet-MUC21 artificial APC assay. (**E**) Representative FACS plots showing the expression of MUC21 in p815-OKT3-tet-MUC21 cells upon Dox (1 µg/ml) treatment. (**F**) Representative FACS plots showing proliferating CTV_low_ CD8^+^T cells (above panel) and a summary graph showing the division index of CTV_low_ CD8^+^T cells (below panel). (**G**) ELISA analysis of IFN-γ secretion by CD8^+^T cells. (**H**-**I**) 1G4 TCR-engineered CD8^+^T (1G4 TCR-CD8^+^T) cells were co-cultured with Raji cells expressing A*02:01/NY157–165 single-chain trimers, PD-L1 and Dox-inducible MUC21 (Raji-A2-ESO-1-PD-L1-MUC21) for three days, in the presence of the indicated antibodies (10 µg/ml) or Dox (1 µg/ml). (**H**) Schematic illustration of 1G4 TCR-engineered CD8^+^T cell-mediated Raji-A2-ESO-1-PD-L1-MUC21 cell killing. (**I**) Percentages of antigen-specific killing of Raji-A2-ESO-1-PD-L1-MUC21 cells by 1G4 TCR-CD8^+^T cells at an E:T ratio of 1:10 in the presence of the indicated antibodies with or without Dox. Data were compiled from three independent experiments with two replications. Statistical significance was determined by two-tailed unpaired *t*-test in (**A**), (**B**), (**C**), (**F**) and (**G**) or one-way ANOVA with Holm-Sidak multiple comparisons in (**I**). *P < 0.05, **P < 0.01, ***P < 0.001; and ns, not significant

NK cells serve as major effectors of ADCC via CD16 (FcγRIII). We investigated whether surface MUC21 expression could render tumor cells resistant to antibody-dependent NK cytotoxicity using NK-92 cells stably expressing human CD16 (NK-92-CD16). Raji-tet-MUC21 cells, which are CD20-positive lymphoma cells stably expressing Dox-inducible MUC21 (Suppl. Figure S3A), were co-incubated with NK-92-CD16 cells with or without rituximab (anti-CD20 antibody). Consistent with the findings in the K562 cell lines, surface MUC21 expression in Raji cells suppressed NK-mediated killing. Rituximab efficiently increased the cytotoxic activity of NK-92-CD16 cells against Dox non-treated Raji-tet-MUC21 cells. However, when MUC21 expression was induced on Raji-tet-MUC21 cells using Dox, this effectively abrogated the ADCC activity of rituximab (Fig. 2A). Consistently, the addition of rituximab resulted in an increased degranulation of NK cells. However, when exposed to Dox-treated Raji-tet-MUC21 cells, there was a notable reduction in NK cell degranulation compared to Dox non-treated Raji-tet-MUC21 cells (Fig. 2B). It was confirmed that MUC21 expression did not hinder the binding of rituximab to the target cells, indicating that the impaired ADCC activity was not related to antibody binding to its target (Suppl. Figure S3B). Based on previous studies that have indicated a high expression of MUC21 in lung cancer [26, 27], we screened for the expression of MUC21 in various lung cancer cell lines using flow cytometry (Suppl. Figure S4). These analyses revealed that NCI-H441 cells exhibit surface expression of MUC21 (Fig. 2C). Notably, the expression of MUC21 was found to be significantly elevated in 3D spheroid cultures of NCI-H441 cells compared to 2D cultures, indicating a potential role for MUC21 in anti-adhesion mechanisms. To then examine whether endogenous MUC21 expression could affect ADCC mediated by NK cells, MUC21 knockdown NCI-H441 cells (sh*MUC21*) were generated with lentivirus expressing *MUC21*-specific shRNA and a FACS-based cell selection (Fig. 2C). shCTL and sh*MUC21* NCI-H441 cells were co-cultured with NK-92-CD16 cells in the presence or absence of cetuximab (anti-EGFR antibody). We observed that NK-92-CD16 cells exhibited slightly higher cytotoxicity against sh*MUC21* NCI-H441 cells compared to the shCTL cells. However, the addition of cetuximab significantly enhanced the cytotoxicity of NK-92-CD16 cells against sh*MUC21* NCI-H441 cells compared to shCTL NCI-H441 cells (Fig. 2D). This finding suggests that inhibiting MUC21 could potentially improve the effectiveness of ADCC-based drugs. We confirmed that the expression of MUC21 did not impede the binding of cetuximab to NCI-H441 cells (Suppl. Figure S3C). An increased surface expression of CD107a was observed on NK-92-CD16 cells upon co-incubation with MUC21 knockdown NCI-H441 cells (Fig. 2E), again pointing to the suppressive function of MUC21. We further examined the inhibitory effects of MUC21 on antibody-mediated NK cytotoxicity using A549 lung cancer cells, which lack endogenous MUC21. These cells were modified to express Dox-inducible MUC21 (Suppl. Figure S3D). Surface expression of MUC21 on A549 cells inhibited NK-induced cytotoxicity. Cetuximab administration augmented the cytotoxic activity of NK cells against Dox non-treated A549-tet-MUC21 cells. Yet, when MUC21 expression was induced on A549-tet-MUC21 cells via Dox treatment, it significantly diminished the ADCC effectiveness of cetuximab (Fig. 2F). The expression status of MUC21 had no effect on the binding of cetuximab to A549 cells (Suppl. Figure S3E). We next examined whether surface MUC21 expression affected the CDC activity of rituximab. CDC is an important mode of action of rituximab, with a significant impact on its clinical efficacy. The CDC assay, mediated by rituximab, showed that the overexpression of MUC21 did not provide protection to Raji cells from complement-mediated lysis (Fig. 3G). Taken together, these findings indicate that surface MUC21 expression may impact cell-mediated cytotoxic activity.

### Surface MUC21 expression inhibits T cell activation and function

We investigated whether MUC21 affects T cell activation. Human primary CD8^+^T cells underwent activation using plate-coated anti-CD3 antibodies while being co-cultured with either 293FT cells or MUC21-expressing 293FT cells as a matrix for surface MUC21 (Suppl. Figure S5A). Following a five day co-culture period, the activation of CD8^+^T cells was assessed by measuring the expression of activation markers, proliferation, and cytokine secretion. The presence of MUC21 resulted in a decreased expression of CD25 and CD69 by CD8^+^T cells (Fig. 3A). Furthermore, CD8^+^T cells exhibited reduced proliferative capacity and IFN-γ secretion when co-cultured with MUC21-expressing 293FT cells (Fig. 3B C). To next examine whether surface MUC21 inhibits T cell activation by interfering with the interaction between T cells and antigen-presenting cells (APCs), we employed p815 cells expressing membrane-bound anti-CD3 single-chain fragment variable (scFv) as artificial APCs (p815-OKT3). To prevent any intrinsic effects of constitutive MUC21 expression on p815 cells, we engineered p815-OKT3 cells to stably express MUC21 in a Dox-dependent manner (p815-OKT3-tet-MUC21; Fig. 3D and E). The Dox-induced MUC21 expression on p815-OKT3 cells hindered efficient T cell activation, resulting in decreased proliferation and IFN-γ secretion, regardless of the relative amount of MUC21 expression (Fig. 3F and G). Based on our findings, we hypothesized that T cell-targeting immunotherapeutics may not effectively activate T cells in the presence of MUC21. To test this hypothesis, we utilized CD8^+^T cells expressing the 1G4 TCR that specifically recognizes the NY-ESO-1 cancer testis antigen in an HLA-A*0201-restricted manner [16]. Raji cells expressing A*02:01/NY157–165 single-chain trimers (Raji-A2-ESO-1) were engineered to stably express constitutive PD-L1 and Dox-inducible MUC21, which enables the assessment of antigen specific CD8^+^T cell cytotoxic activity upon treatment with anti-PD-(L)1 checkpoint inhibitors in the presence of MUC21 (Fig. 3H and S5B). Treatment with Dox resulted in a reduced killing rate of Raji-A2-ESO-1-PD-L1-MUC21 cells by 1G4 TCR-CD8^+^T cells compared to untreated cells. This decreased cytotoxic activity of 1G4 TCR-CD8^+^T cells was not restored by anti-PD-L1 blocking. Furthermore, a directly triggering co-stimulatory signal with anti-4-1BB agonist antibodies did not compensate for the MUC21-mediated inhibition of 1G4 TCR-CD8^+^T cell responses (Fig. 3I). In summary, these results indicated that MUC21 plays a crucial role as a negative regulator of T cell activation by hindering the antigen engagement of T cells.

### Surface MUC21 expression inhibits the cytotoxic activities of anti-CD19-CAR-T and CAR-NK cells

**Fig. 4:**
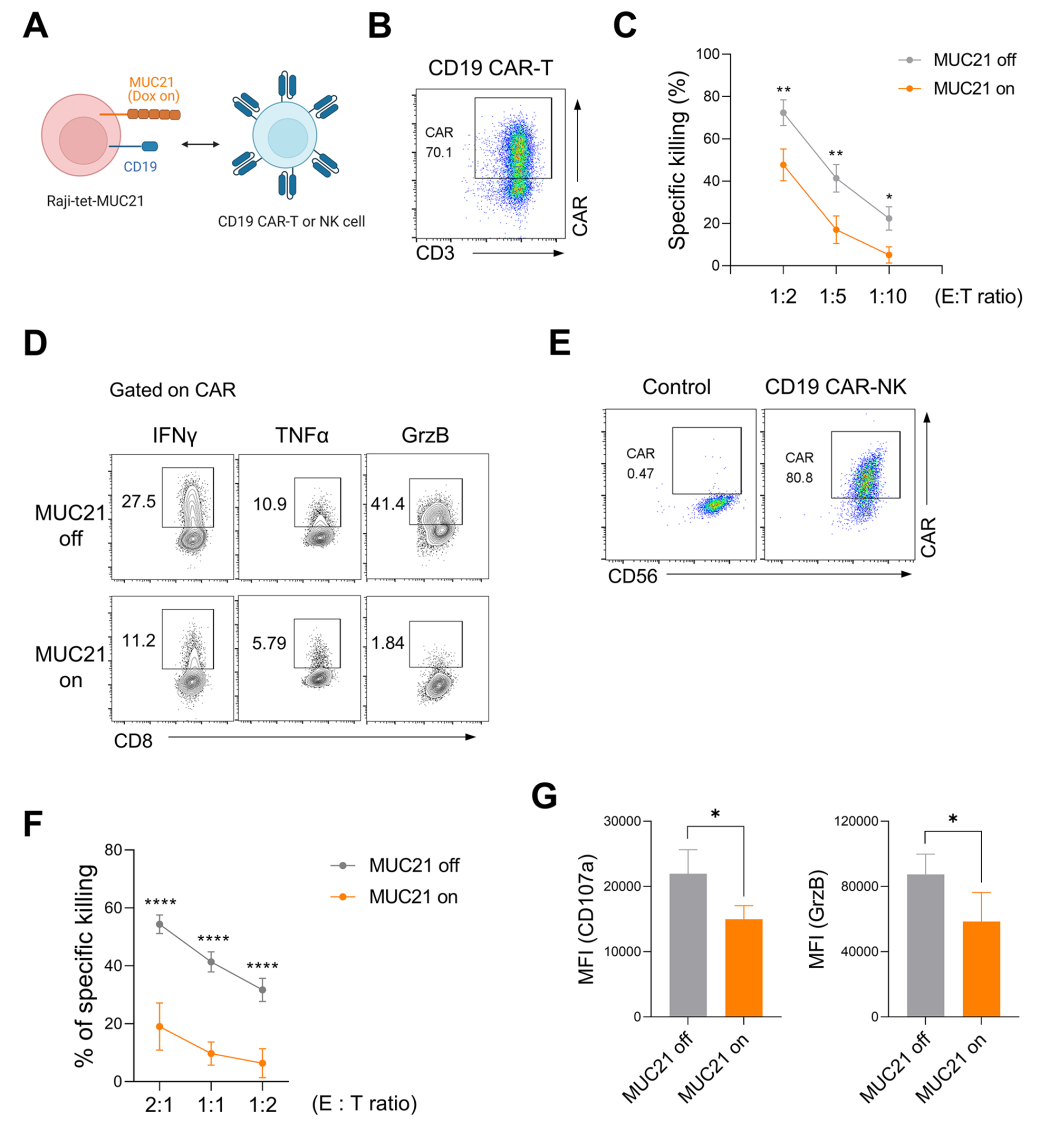
Expression of MUC21 on the cell membrane suppresses the cytotoxic functions of anti-CD19 CAR-T and CAR-NK cells. (**A**-**D**) CD3^+^T cells isolated from human PBMCs were stimulated with anti-CD3/CD28 beads and transduced with anti-CD19 CAR lentivirus. CD19 CAR-T cells were co-incubated with Raji-tet-MUC21 cells at variable E:T ratios for two days in the presence or absence of Dox. (**A**) Schematic illustration of the killing of Raji-tet-MUC21 cells by CD19-CAR T or NK cells. (**B**) FACS analysis of the expression of anti-CD19 CAR on T cells using biotinylated CD19 protein. (**C**) The percentages of killing of Raji-tet-MUC21 cells by CD19 CAR-T cells were determined at the indicated E:T ratios. (**D**) Representative FACS plots showing the percentages of IFN-γ, TNF-α or Granzyme B (GrzB) expression by CD19 CAR-T cells co-incubated with Raji-tet-MUC21 cells at an E:T ratio of 1:2. (**E**-**G**) NK-92 cells were transduced with anti-CD19 CAR lentivirus. CD19 CAR-NK-92 cells were co-incubated with Raji-tet-MUC21 cells at variable E:T ratios for four hours in the presence or absence of Dox (1 µg/ml). (**E**) FACS analysis of the expression of anti-CD19 CAR on NK-92 cells using biotinylated CD19 protein. (**F**) Percentages of the specific killing of Raji-tet-MUC21 cells by CD19 CAR-NK-92 cells at the indicated E:T ratios. (**G**) Summary graph showing the mean fluorescence intensity (MFI) of CD107a and GrzB expression in CD19 CAR-NK-92 cells co-incubated with Raji-tet-MUC21 cells at an E:T ratio of 1:1. Data were compiled from four independent experiments with two replicates. Statistical significance was determined by a 2-way ANOVA with Holm-Sidak comparisons in (**C**) and (**F**), or two-tailed unpaired *t*-tests in (**G**). *P < 0.05, **P < 0.01, ****P < 0.0001

CAR expression renders T cells to be more responsive to antigen stimulation through its intracellular costimulatory domains. In addition, CARs generally have a higher affinity for antigen compared to TCRs. We thus investigated whether CAR-T cells can overcome the inhibitory effects of MUC21 on antigen engagement (Fig. 4A and B). To assess this, we incubated CD19 CAR-T cells, generated by transducing human CD3^+^T cells with anti-CD19 CAR lentivirus, with Raji-tet-MUC21 cells. We observed that when the expression of MUC21 was induced, there was a nearly two-fold reduction in the killing rate of Raji-tet-MUC21 cells at various E:T ratios, as compared to the absence of MUC21 expression (Fig. 4C). This decrease in cytotoxic activity was accompanied by a suppression of cytotoxic cytokine production, including IFN-γ, TNF-α, and granzyme B, in CD19 CAR-T cells upon MUC21 expression on target cells (Fig. 4D). These findings indicate that CAR-T cells are unable to effectively utilize their intracellular costimulatory domains in the presence of MUC21. We further assessed the cytotoxic activity of CAR-NK-92 cells against MUC21-expressing cancer cells to evaluate whether surface MUC21 exerts a similar inhibitory effect on CAR-NK cell responses. NK-92 cells were engineered to express anti-CD19-CAR (Fig. 4E). Dox-induced MUC21 expression on Raji cells showed a substantial resistance to killing by CD19 CAR-NK-92 cells (Fig. 4F), as seen in the cytotoxic activity of CAR-T cells. This attenuated cytotoxicity correlated with the decrease of cell surface CD107a and granzyme B expression in CD19-CAR-NK-92 cells (Fig. 4G). Collectively, these results suggested that surface MUC21 expression blunts the sensitivity of CAR antigen recognition by both T and NK cells.

### Surface MUC21 expression on cancer cells blocks their interaction with immune cells

**Fig. 5:**
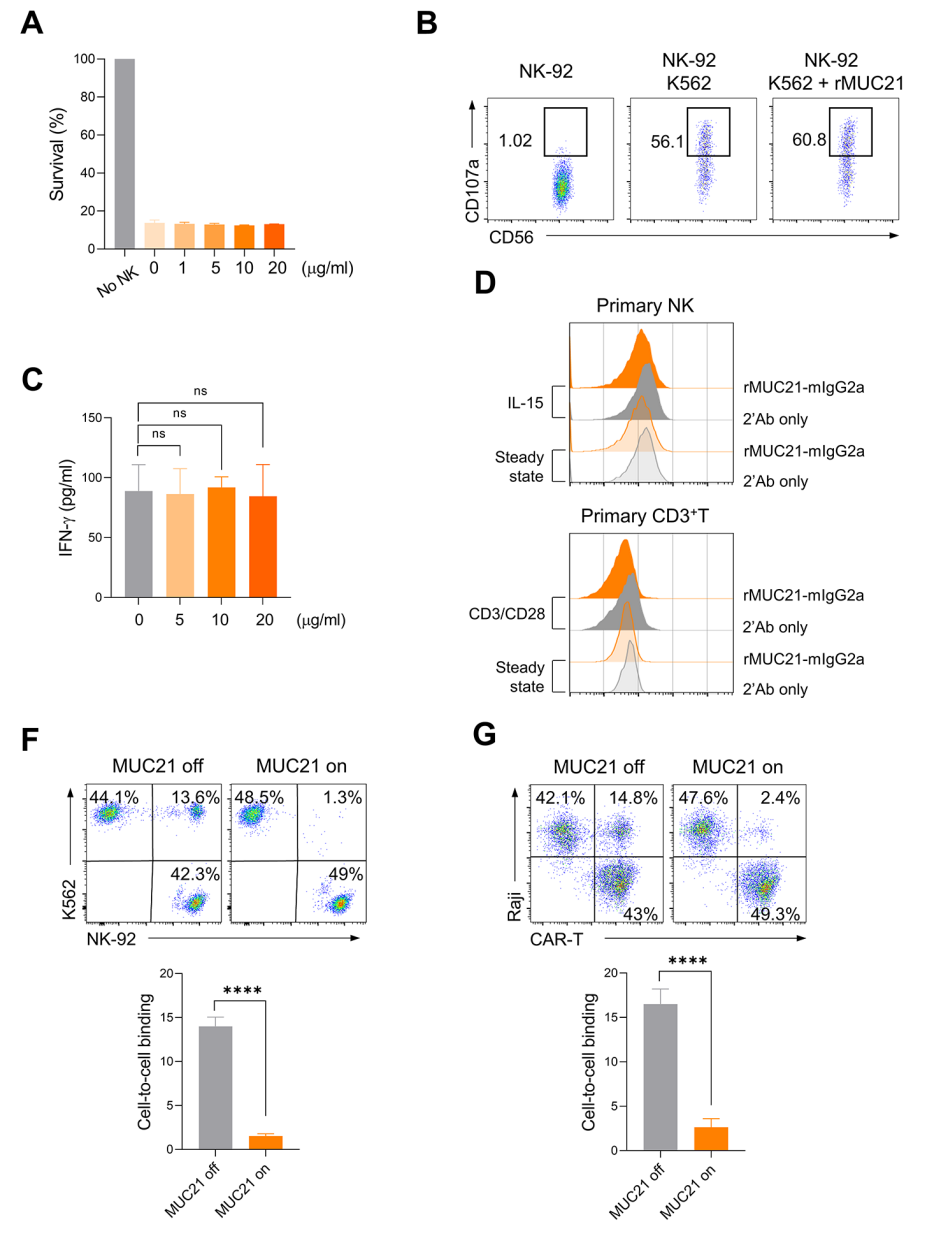
The presence of membrane-bound MUC21 on cancer cells obstructs their interaction with immune cells. (**A**-**B**) K562 cells were co-cultured with NK-92 cells at an E:T ratio of 0.5:1 for four hours in the presence of varying concentrations of rMUC21-mFc. (**A**) The cytotoxicity against K562 cells was measured by the luciferase activity of the surviving cells. (**B**) Representative flow cytometry analysis of surface CD107a expression on NK cells. (**C**) Human CD8^+^T cells were activated with anti-CD3 antibody in the presence of varying concentrations of rMUC21-mFc. ELISA of IFN-γ secretion by CD8^+^T cells. (**D**) FACS analysis showing the binding of rMUC21-mFc to resting and IL-15-stimulated primary NK cells (above), as well as resting and activated primary CD3^+^ T cells (below). (**F**) K562-tet-MUC21 cells were cultured in the presence or absence of Dox for 24 h. These cells were then co-incubated with CTV-labeled NK-92 cells for 15 min. Representative FACS plots (above) and a summary plot (below) showing the percentages of cell-to-cell conjugation. (**G**) Raji-tet-MUC21 cells were co-incubated with CTV-labeled CD19 CAR-T cells for 15 min. Representative FACS plots (above) and a summary plot (below) showing the percentages of the cell-to-cell binding. Data were compiled from two independent experiments. Statistical significance was determined by two-tailed unpaired *t*-tests. ns: not significant. ****P < 0.001

Given that certain MUC family proteins, such as MUC1, MUC4, and MUC16, can be shed from the cell surface and released into the extracellular space in soluble forms [28], we examined whether a soluble form of MUC21 can inhibit the antitumor activities of immune cells. To generate soluble MUC21 proteins, the extracellular domain of MUC21 (amino acids 25–479) was fused to the Fc domain of mouse IgG2a (rMUC21-mFc). We first investigated whether the recombinant soluble MUC21 protein affects the cytotoxic activity of NK-92 against K562 cells. As shown in Fig. 5A, NK-92 cells efficiently killed K562 cells even in the presence of a high concentration of rMUC21-mFc. Treatment with rMUC21-mFc also had no effect on the expression of surface CD107a in NK cells (Fig. 5B). We further examined the influence of soluble MUC21 protein on T cell activation. The presence of rMUC21-mFc did not affect the levels of IFN-γ production by CD8^+^T cells stimulated with anti-CD3 antibody (Fig. 5C). Collectively, these results suggested that the soluble form of MUC21 does not influence NK cytotoxicity or T cell activation, in contrast to its membrane-bound form. Certain mucin family proteins, such as MUC1 and MUC16, have been reported to interact with cell surface receptors on immune cells, thereby modulating the function of these cells [21]. Hence, we examined whether T cells and NK cells express surface receptors capable of interacting with the extracellular domain of MUC21. This binding experiment revealed that rMUC21-mFc did not bind to either resting or activated primary T cells, nor to resting or activated primary NK cells (Fig. 5D). Based on these observations, it is unlikely that the immune suppressive function of MUC21 depends on the interaction between membrane-bound MUC21 and specific receptors on T and NK cells.

Despite its short cytoplasmic tail of 49 amino acids [23], MUC21 has been reported to be associated with the STAT3/AKT and hedgehog pathways in glioblastoma and melanoma, respectively, leading to tumor cell proliferation and migration [29, 30]. We thus investigated whether MUC21 overexpression triggers diverse transcriptional changes in cancer cells, thus enabling their immune evasion. Global gene expression analysis was performed on Raji-tet-MUC21 cells treated with or without Dox. Upon comparing the MUC21 high-expressing cells with the control cells, a total of 45 upregulated and 38 downregulated differentially expressed (DE) genes were identified, meeting the criteria of an absolute fold change > 2 and a *P*-value < 0.01 (Suppl. Figure S6). However, due to the small number of DE genes and their distribution across different pathways, we were unable to obtain statistically significant results in our subsequent pathway analysis using PANTHER (83.3% unclassified PANTHER category) and GSEA (no enriched pathway with FDR < 0.05) (data not shown). Surface mucin proteins have been found to impact the invasive and metastatic traits of cancer cells by modulating cell adhesion and anti-adhesion mechanisms [31]. Therefore, we investigated whether the presence of membrane-bound MUC21 on cancer cells affects their interaction with immune cells. NK-92 cells were labeled with CellTrace Violet dye and co-incubated with K562-tet-MUC21 cells, which constantly express RFP in a bicistronic manner. Flow cytometry was employed to assess the binding between these two cell types, identified by the occurrence of events showing double positivity. Our results revealed that non-Dox treated K562-tet-MUC21 cells exhibited binding with NK-92 cells, whereas MUC21 expression induced by Dox significantly hindered this interaction (Fig. 5F). Furthermore, we replicated these findings using CD19 CAR-T cells and CD19-expressing Raji B cells. In this case, the expression of MUC21 in Raji-tet-MUC21 cells effectively suppressed their interaction with CD19 CAR-T cells (Fig. 5G). Taken together, these findings suggested that the presence of MUC21 on the surface of cancer cells can impede the cytotoxic function of NK and T cells by blocking their interaction with the cancer cells.

### High *MUC21* expression is associated with reduced cytotoxicity and anti-PD-(L)1 resistance in LUAD

**Fig. 6:**
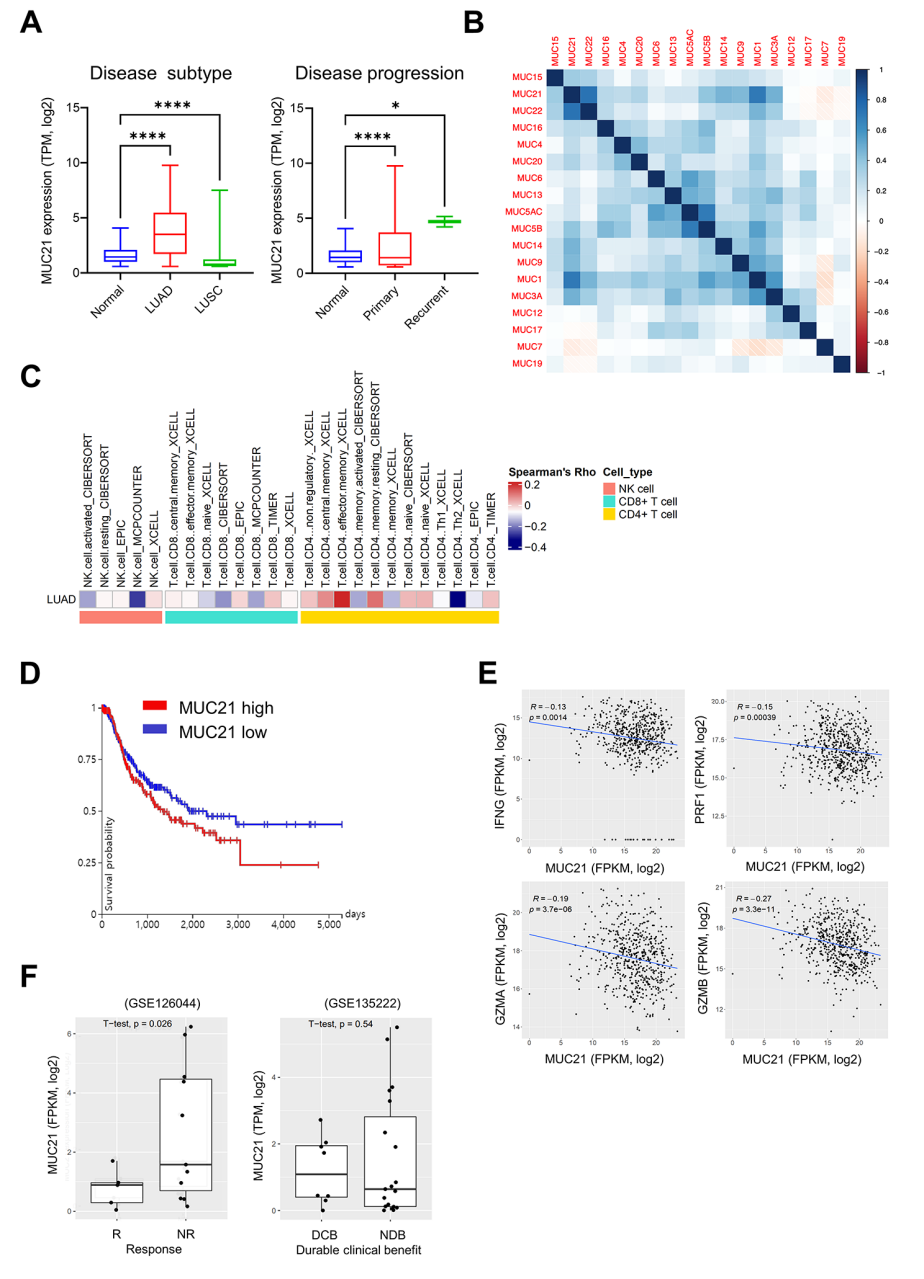
Negative correlation between elevated MUC21 expression and immune cytotoxicity in LUAD. (**A**) Expression levels of the *MUC21* gene in LUAD and LUSC patients from TCGA, along with their matched normal individuals from TCGA and GTEx. This analysis considered both the disease subtype and progression. Statistical significance was assessed using one-way ANOVA and a Dunnett’s multiple comparison test. (**B**) Heatmap illustrating the Pearson correlation coefficients between the gene expression levels of each member of the mucin family in TCGA LUAD samples. (**C**) Heatmap displaying scores representing the infiltration of NK cells, CD8^+^T cells, and CD4^+^T cells based on different immune cell deconvolution methods in TCGA LUAD. (**D**) Progression-free survival curve of lung cancer patients (LUAD and LUSC) from TCGA plotted based on the expression of the *MUC21* gene. The Kaplan-Meier curve compares the top and bottom quartiles of *MUC21* expression, and significance was evaluated using log-rank test statistics. (**E**) Scatter plots depicting the correlations between the expression of the *MUC21* gene and cytotoxicity genes (*IFNG*, *PRF1*, *GZMA*, and *GZMB*) involved in NK/T cell-mediated cytotoxic responses in TCGA LUAD. The correlation was tested using a Spearman’s rank correlation coefficient. (**F**) Boxplots comparing the expression of the *MUC21* gene between patients who responded to anti-PD-(L)1 immune checkpoint inhibitor therapy and those who did not respond in two different lung cancer cohorts. Responders (R) or those with durable clinical benefit (DCB) achieved partial response (PR) or stable disease (SD) for more than six months. Non-responders (NR) or those with non-durable benefit (NDB) experienced progressive disease (PD) or SD for less than six months. The significance of the differences was evaluated using a Student’s *t*-test

To further explore the clinical implications of MUC21 for cancer cell immunity, we analyzed its expression in various tumor types in TCGA cancer patients and corresponding normal tissues from GTEx. We observed aberrantly high expression of *MUC21* in several tumor types, including lung adenocarcinoma (LUAD), cervical squamous cell carcinoma (CESC), and thyroid carcinoma (THCA) (Suppl. Figure S7). Furthermore, the expression of *MUC21* was found to be higher in recurrent LUAD compared to primary LUAD (Fig. 6A). In addition, immortalized cancer cell lines derived from the lung, head and neck, and cervix also exhibited elevated expression of *MUC21* (Suppl. Figure S8). Analysis of the methylation status of the *MUC21* promoter region indicated that the aberrant overexpression of *MUC21* in LUAD could be attributed to epigenetic regulation (Suppl. Figure S9). Considering the high expression of certain members of the MUC family in cancer cells [32], we investigated the correlation between the expression levels of each MUC family member in TCGA NSCLC. We observed a strong correlation between *MUC21* and *MUC22*, with MUC22 ranking as the third top hit among our CRISPRa screening results (Figs. 1B and 6B). The co-localization of *MUC22* and *MUC21* in the mucin gene cluster on chromosome 6p21.3 suggests a link between these genes in lung cancer progression and heterogeneity [33, 34]. Additionally, we found a high correlation between *MUC21* and *MUC1* expression, which is known for its immune-suppressive role in NSCLC. MUC family members play a crucial role in forming physical protective barriers against molecules and microbes. By employing diverse deconvolution algorithms, we thus analyzed the bulk RNA-sequencing data from TCGA LUAD to determine the subtypes of intratumoral NK and T cell infiltration. The results revealed a negative correlation between *MUC21* expression and the infiltration of activated NK cells and CD8^+^T cells, while no significant association was found with CD4^+^T cells (Fig. 6C). To evaluate the potential association between reduced immune cell infiltration and the prognosis of NSCLC patients, we analyzed the progression-free survival data for the TCGA NSCLC samples, stratifying them based on *MUC21* expression. This analysis revealed that the high quartile group of *MUC21* expression (n = 250) exhibited a poorer prognosis compared to the low quartile group (n = 252), although statistical significance was not reached (*P* = 0.127; Fig. 6D). In the subsequent analysis, we explored the correlation between *MUC21* expression and the expression of cytotoxicity genes involved in NK cell and CD8^+^T cell-mediated antitumor responses among the NSCLC patients in the TCGA dataset. *MUC21* expression showed significant negative correlations with *IFNG* (R = -0.13, *P* = 0.0014), *PRF1* (R = -0.15, *P* = 0.00039), *GZMB* (R = -0.27, *P* = 3.3 × 10–11), and *GZMA* (R = -0.19, *P* = 3.7 × 10 − 6), indicating that upregulated *MUC21* correlates with a decreased cytotoxic activity of NK and CD8^+^T cells in NSCLC patients (Fig. 6E). Finally, we investigated the influence of increased *MUC21* expression on the responsiveness of anti-PD-(L)1 immune checkpoint inhibitors. In a retrospective study, we analyzed RNA sequencing gene expression data from two separate cohorts of NSCLC patients treated with anti-PD-(L)1 immune checkpoint inhibitors [35–37]. The analysis revealed that all individuals who responded to the treatment exhibited significantly lower levels of *MUC21* expression (Fig. 6F). Conversely, in the non-responder group, the expression of *MUC21* varied, ranging from high to low depending on the individual. Within the GSE126044 and GSE13522 cohorts, 31.3% (5/16) and 18.5% (5/27) of non-responders, respectively, displayed higher levels of *MUC21* compared to the responders (Suppl. Figure S10). These findings underscore the potential utility of elevated *MUC21* expression as a predictive marker for unresponsiveness to anti-PD-(L)1 inhibitors in LUAD.

## Discussion

Immunosuppressive ligands present on cancer cells engage inhibitory receptors on immune cells and thereby shield the cancer cells from immune system recognition and eradication. In this present study, we conducted gain-of-function screens focused on membrane-bound proteins to identify cancer cell surface ligands that confer resistance to NK cell cytotoxicity. Considering that the loss of a specific immunosuppressive ligand can be compensated by other molecules, employing a gain-of-function approach (CRISPRa) rather than a loss-of-function approach (CRISPR knockout) for discovering immunosuppressive ligands can enhance the efficiency of screening. In addition, a loss-of-function approach fails to capture genes that are not endogenously expressed in given cell types. We have now identified several genes that can suppress NK cell-mediated killing, which helps us to better understand the intrinsic mechanisms of tumor immune resistance and identify potential therapeutic targets.

MUC21 is a high molecular weight glycoprotein that functions as a transmembrane mucin and contains a tandem repeat (TR) domain [23]. Its expression has been observed in various human neoplasms and is associated with the aggressive behavior of neoplastic cells [24, 34, 38, 39]. Several studies have highlighted the role of MUC21 as a negative regulator of cell adhesion, primarily mediated by its glycosylated TR domain [40]. The biological activities of MUC21, including its involvement in the progression of EGFR-mutated lung adenocarcinomas [27] or its ability to confer resistance to apoptosis [41], are thought to rely on specific glycosylation patterns. Although the precise role of MUC21 in tumor-immune interactions remains poorly understood, a recent study has reported that this mucin negatively affects macrophage-mediated antibody-dependent cellular phagocytosis (ADCP) through its anti-adhesion properties against macrophages and antibodies [14].

Our present study findings have revealed that MUC21 expression by cancer cells attenuates NK cell mediated ADCC by inhibiting the conjugation between cancer and NK cells without affecting antibody binding. This suggests that depleting MUC21-expressing cancer cells could be a plausible strategy for improving the efficacy of immunotherapy and overcoming resistance to targeted therapies, particularly in patients with NSCLC. Given the anti-adhesion function of MUC21, it will be important to explore therapeutic strategies that do not rely on immune cell-mediated cytotoxicity. One such promising approach is the use of antibody drug conjugates (ADCs) [42, 43]. When developing an antibody to target MUC21, an essential consideration will be the selection of an epitope that can have a significant impact on the clinical effectiveness of the antibody, as seen in the case of other therapeutic antibodies targeting MUC1 [21]. It has been reported that monoclonal antibodies that target the epitopes in the variable tandem repeat region (VNTR) of the N-terminus of MUC1 (MUC1-N) are ineffective in clinical trials. This lack of efficacy is thought to be due to the shedding of MUC1-N from the cell surface, which results in neutralization of the MUC1 antibodies by the free MUC1-N [44, 45]. Because mucin family proteins share a similar structure in their N-terminus tandem repeat regions, including MUC21 [40], it is also highly likely that MUC21 is shed from cancer cell surfaces. Various strategies have been employed to selectively target specific the glycoforms or domains of mucins [46, 47], which could be potentially applied for engineering the MUC21 antibody. Although the specific glycoforms of MUC21 that are relevant in normal and malignant tissue are not yet well understood, a prior study has reported that an O-glycan recognizing MUC21 antibody only binds to the luminal side of esophageal squamous epithelial cells, but not to carcinoma cells [48]. Hence, targeting these glycoforms with the MUC21 antibody could be a promising future therapeutic strategy.

In addition to the ADC approach, another potential strategy is to exploit the cancer-specific glycoform of MUC21 for engineered immune cell therapies, such as CAR-T cell therapy targeting the cancer associated Tn (GalNAca1-O-Ser/Thr)-glycoform of MUC1 to control various adenocarcinomas [47]. However, unlike MUC1, MUC21 directly inhibits the cell-to-cell contact dependent cytotoxicity of NK or T cells, and a discrete engineering strategy may therefore be required to enable cytotoxic immune cells to circumvent MUC21-mediated anti-adhesion effects.

Apart from its function in modulating NK cell responses, we have here uncovered a novel effect of MUC21 on T cell activation. The forced expression of MUC21 by artificial antigen-presenting cells resulted in an impairment of the initial activation and subsequent effector functions of T cells. Based on its anti-adhesion activity, MUC21 is likely to exert this inhibitory effect on T cell activation by hindering antigen recognition by these cells, rather than triggering inhibitory signals via binding to a specific receptor. Our current data showing no binding of recombinant MUC21 protein to NK or T cells support this hypothesis. The steric hindrance-mediated inhibition of TCR and peptide-MHC engagement by MUC21 allows cancer cells to evade T cell mediated immune surveillance, which may help explain why MUC21 is expressed in incohesive-type lung adenocarcinoma [26] that is prone to metastasis [27, 38]. The prerequisite for effective ICI therapies, such as the administration of anti-PD-1/PD-L1 antibodies, is the prior recognition of tumor antigens by tumor infiltrating T cells. However, the presence of MUC21 prevents T cells from recognizing antigens, thereby limiting the anti-tumor efficacy of ICIs. As we have demonstrated here, treatment with anti-PD-L1 blocking antibodies could not restore the reduced cytotoxic activity of 1G4 TCR CD8^+^T cells against cancer cells expressing MUC21. The defect of CD8^+^T cell responses caused by MUC21 was also not resolved by triggering a strong costimulatory signal with anti-4-1BB agonist antibodies. In addition, even CD19 CAR-T cells, which recognize surface protein antigen and have integrated intracellular costimulatory domains, failed to efficiently confer cytotoxicity in the presence of MUC21. These observations suggest that MUC21 plays a crucial role in tumor immune evasion by blinding T cells to tumor antigens, which may be considered a key tumor cell-intrinsic mechanism of immune evasion [49]. These evasion mechanisms include both loss of tumor antigenicity and immunogenicity, such as impaired antigen presentation and increased expression of immune inhibitory factors [50]. MUC21-mediated immune evasion is thought to solely rely on steric hindrance through its highly glycosylated TR domain [40] rather than via a direct modulation of the intrinsic signaling of cancer cells, since there are no reported intracellular activities of MUC21. This unique mechanism supports the non-redundant role of MUC21 in cancer immune surveillance as it is distinct from MUC1, which shows a highly correlated expression with MUC21 in NSCLC and has an intracellular activity in cancer cells [51]. Given these characteristics of MUC21, developing blocking antibodies would be an impractical approach to its control. Instead, depleting the cancer cells that express MUC21 using the ADC approach, as we have here proposed, would likely be more effective strategy for both targeted treatments and immunotherapies.

## Conclusions

We have here identified MUC21 as an immunosuppressive ligand that obstructs NK cell cytotoxicity and inhibits T cell activation. Targeting MUC21 could present an opportunity to enhance the effectiveness of immunotherapy and overcome resistance in NSCLC by circumventing the immune evasion mechanisms employed by cancer cells.

### Electronic supplementary material

Below is the link to the electronic supplementary material.

Supplementary Material 1

## Data Availability

The data that support the findings of this study are available from the corresponding author upon request.

## Abbreviations

ADC: Antibody drug conjugate
ADCC: Antibody-dependent cell-mediated cytotoxicity
ADCP: Antibody-dependent cellular phagocytosis
APC: Antigen-presenting cell
BFP: Blue fluorescent protein
CAR: Chimeric antigen receptor
CDC: Complement-dependent cytotoxicity
CRISPR: Clustered regularly interspaced short palindromic repeats
EGFR: Epidermal growth factor receptor
GZMA: Granzyme A
GZMB: Granzyme B
HLA: Human leukocyte antigen
ICI: Immune-checkpoint inhibitor
IFN-γ/IFNG: Interferon gamma
IgG: Immunoglobulin G
IL: Interleukin
MHC: Major histocompatibility complex
PBMC: Peripheral blood mononuclear cell
PD-1: Programmed cell death protein 1
PD-L1: Programmed Death-Ligand 1
STAT3: Signal transducer and activator of transcription 3
TCR: T cell receptor
TNF-α: Tumor necrosis factor-α
TR: Tandem repeat
VNTR: Variable number tandem repeat
