# Supplementary material for: A CRISPR activation screen identifies MUC-21 as critical for resistance to NK and T cell-mediated cytotoxicity
Source: J Exp Clin Cancer Res. 2023 Oct 20;42:272. doi: 10.1186/s13046-023-02840-9 (PMC10588101; doi:10.1186/s13046-023-02840-9)
Supplement: Supplementary file 1 — Supplementary Material 1 [file 13046_2023_2840_MOESM1_ESM.docx]

**
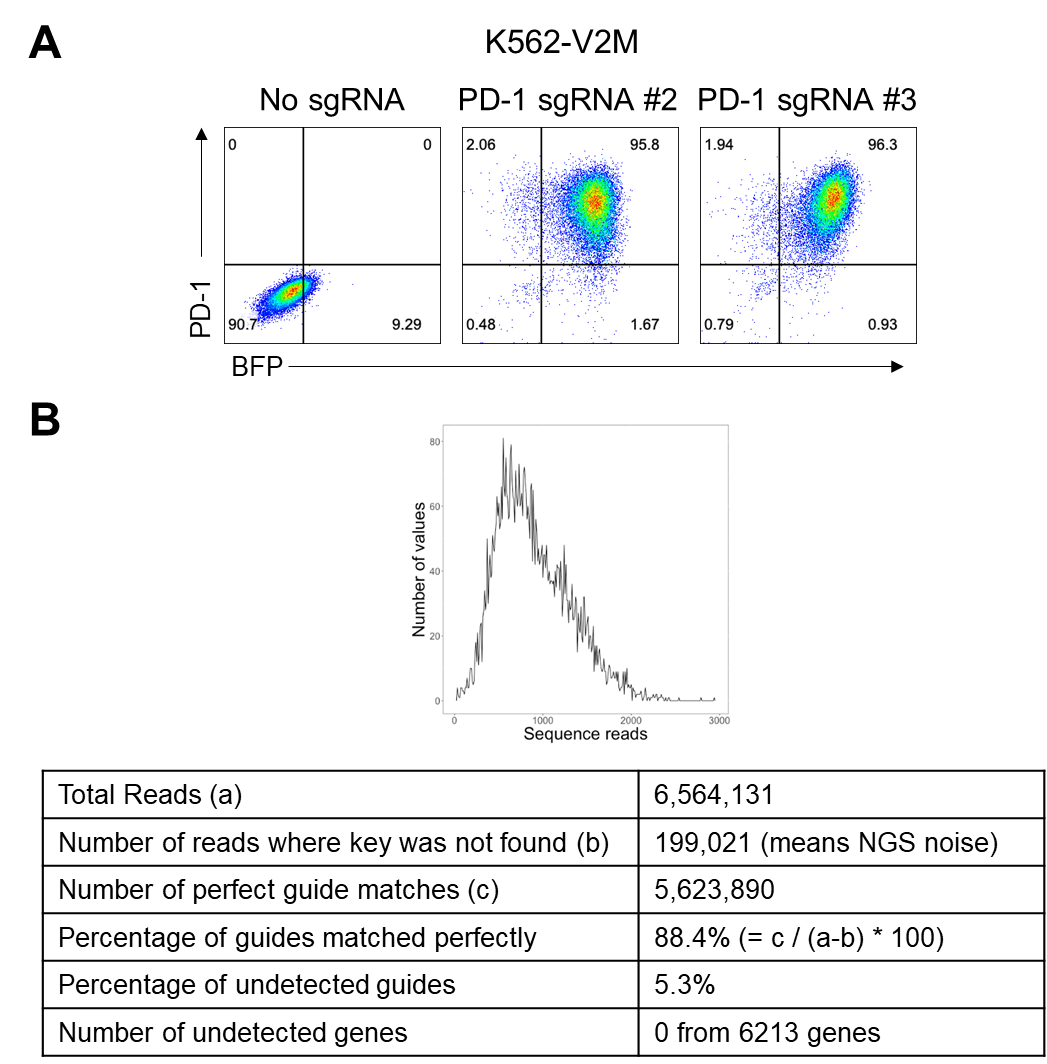
**

**Suppl. Figure S1. A CRISPR activation sgRNA library targeting cell surface receptors.**

(A) Validation of a single K562 cell clone expressing VP64-dCas9-VP64 and MS2-p65-HSF1 (K562-V2M). Lentivirus was used to transduce K562-V2M cells with sgRNA targeting the promoter regions of *PDCD-1*. FACS analysis assessing the upregulation of PD-1 in K562-V2M cells.

(B) Distribution analysis of sgRNA in the Wright Human Membrane Protein Activation Library. The amplified sgRNA library underwent sequencing using an Illumina NGS sequencer (above plot). The resulting FASTQ file was analyzed using a Python script. The count values for each sgRNA were plotted using a polygon plot, based on a total of 6,564,131 reads. Statistical analysis of the library revealed that 88.4% of the guides had perfect matches, while 5.3% of the guides were not detected (below plot).

**
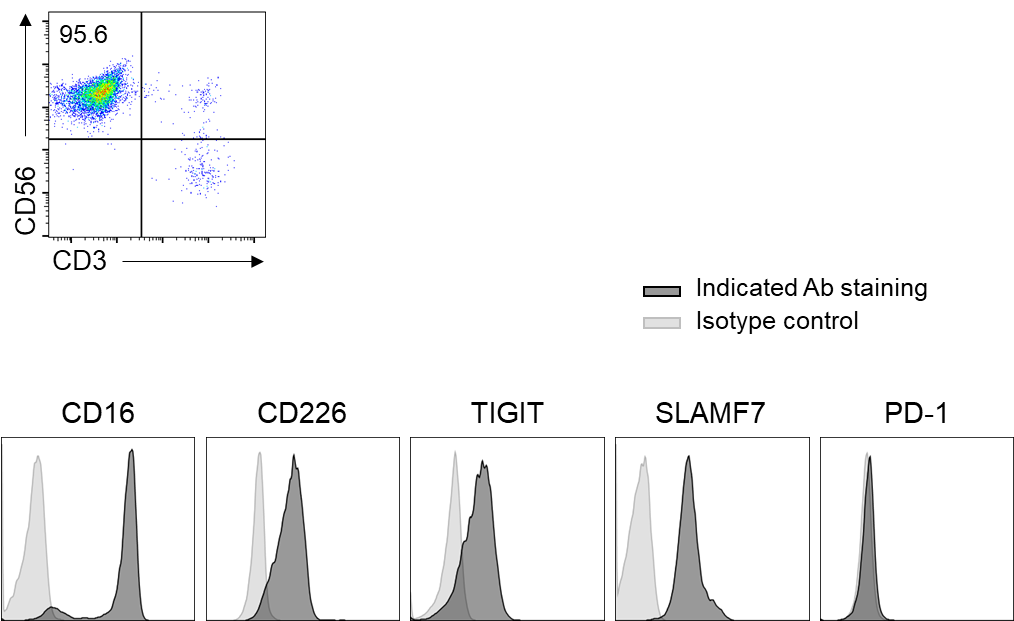
**

**Suppl. Figure S2. Immunophenotype of expanded primary human NK cells.**

Freshly prepared peripheral blood mononuclear cells (PBMCs) were co-cultured with γ-irradiated K562 feeder cells on day 0 in the presence of 10 U/mL of IL-2. Re-stimulation was performed on days 7 and 14 using 100 U/mL of IL-2 and 5 ng/mL of IL-15. (A) The purity of the expanded NK cells was assessed using flow cytometry. (B) The expression of surface receptors on the expanded primary human NK cells was analyzed, including CD16, CD226, TIGIT, SLAMF7 and PD-1

**
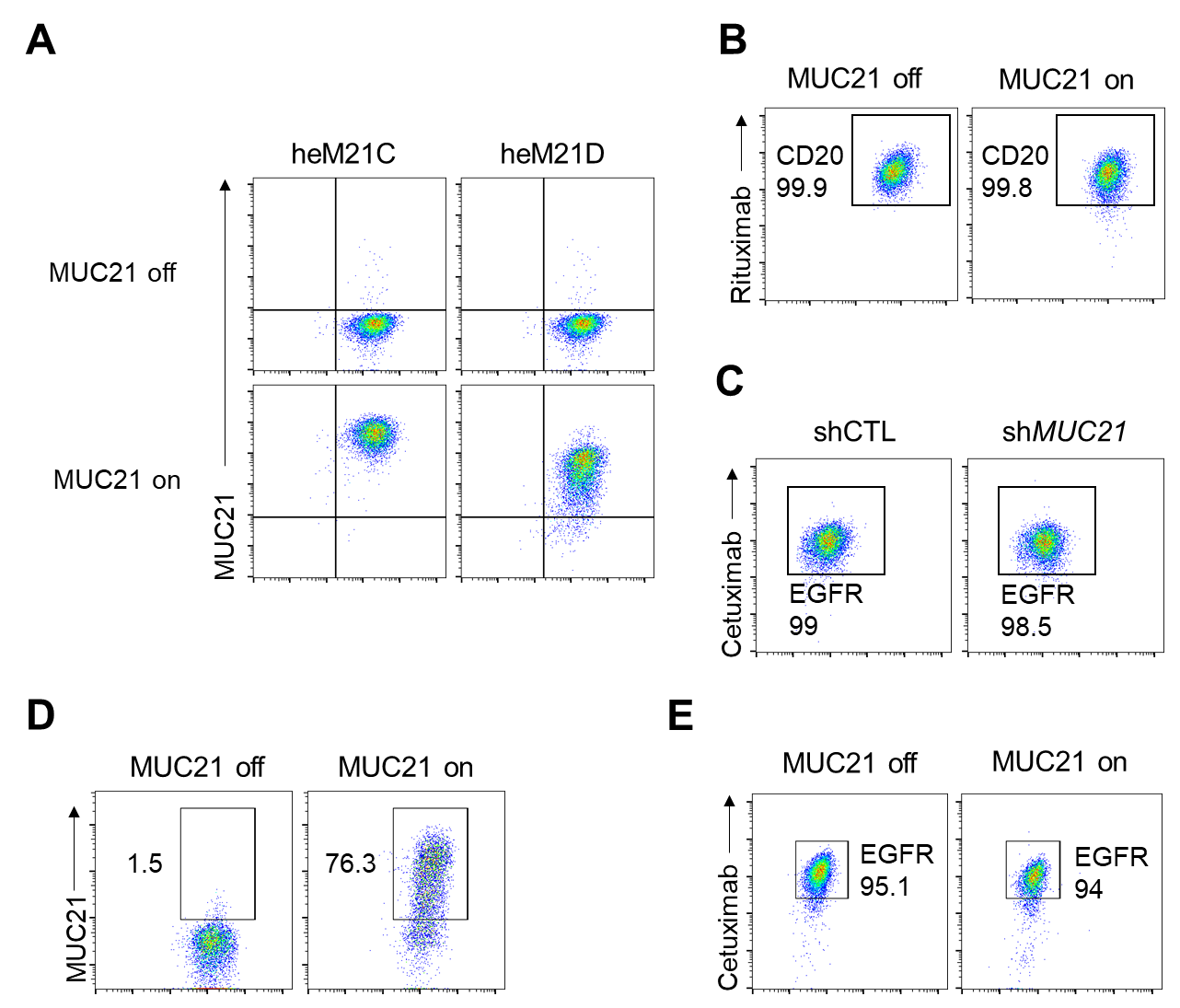
**

**Suppl. Figure S3.** (A) Flow cytometric analysis of MUC21 expression in Raji-tet-MUC21 cells treated with 1 μg/ml Dox (MUC21 on) or without Dox (MUC21 off) for 24 hours. The cells were stained with anti-heM21C or anti-heM21D antibodies. (B) Flow cytometric analysis of rituximab binding to Raji-tet-MUC21 cells treated with 1 μg/ml Dox (MUC21 on) or without Dox (MUC21 off) for 24 hours. (C) Flow cytometric analysis of cetuximab binding to shCTL and sh*MUC21* NCI-H441 cells. (D) Flow cytometric analysis of MUC21 expression in A549-tet-MUC21 cells treated with 1 μg/ml Dox (MUC21 on) or without Dox (MUC21 off) for 24 hours. (E) Flow cytometric analysis of cetuximab binding to A549-tet-MUC21 cells treated with 1 μg/ml Dox (MUC21 on) or without Dox (MUC21 off) for 24 hours.


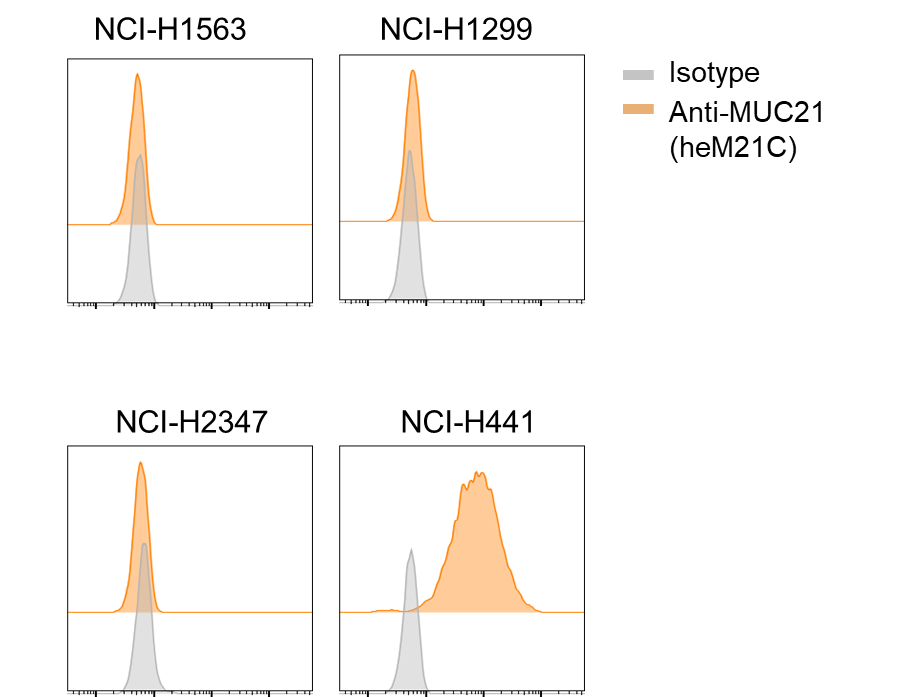


**Suppl. Figure S4.** (A) Flow cytometric analysis of MUC21 expression in lung cancer cell lines. The cell line NCI-H1563, NCI-H1299, NCI-H2347, and NCI-H441 cells were stained with either isotype control antibody or anti-heM21C antibody, followed by treatment with an APC-conjugated anti-mouse secondary antibody.


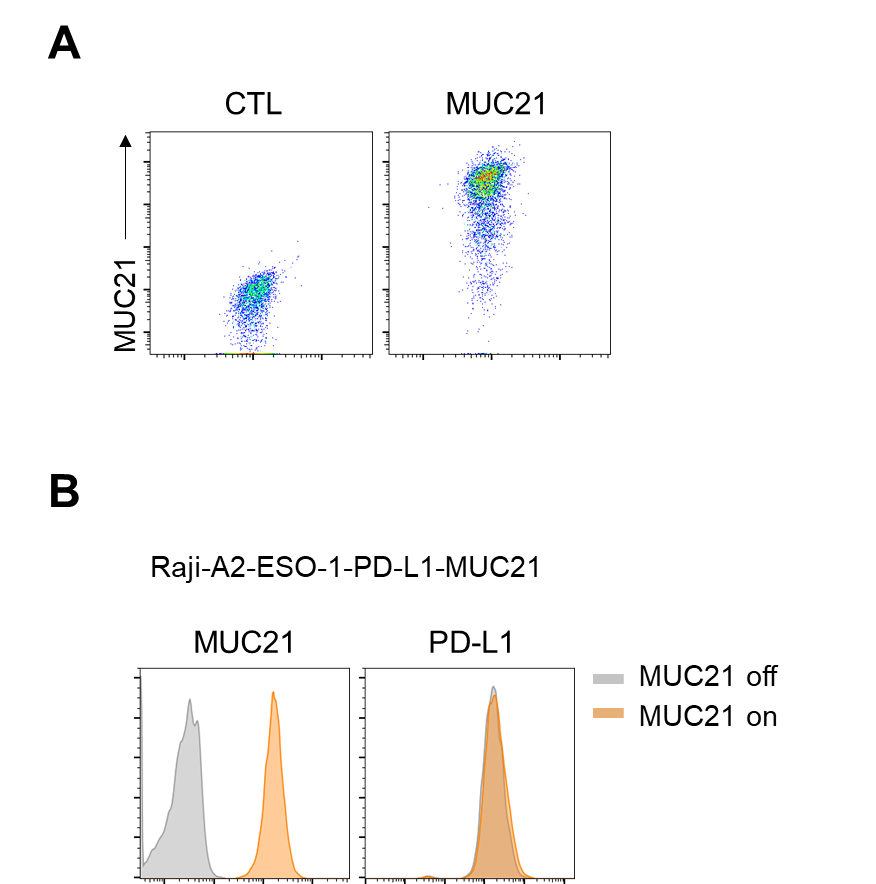


**Suppl. Figure S5.** (A) Flow cytometric analysis of MUC21 expression in 293FT cells transfected with a plasmid encoding human *MUC21*. The cells were stained with anti-Myc antibodies. (B) Flow cytometric analysis of the expression of MUC21 and PD-L1 in Raji-A2-ESO-1-PD-L1-MUC21 cells. The cells were treated with 1 μg/ml Dox (MUC21 on) or without Dox (MUC21 off) for 24 hours, subsequently stained with anti-PD-L1 and anti-Myc antibodies.

**
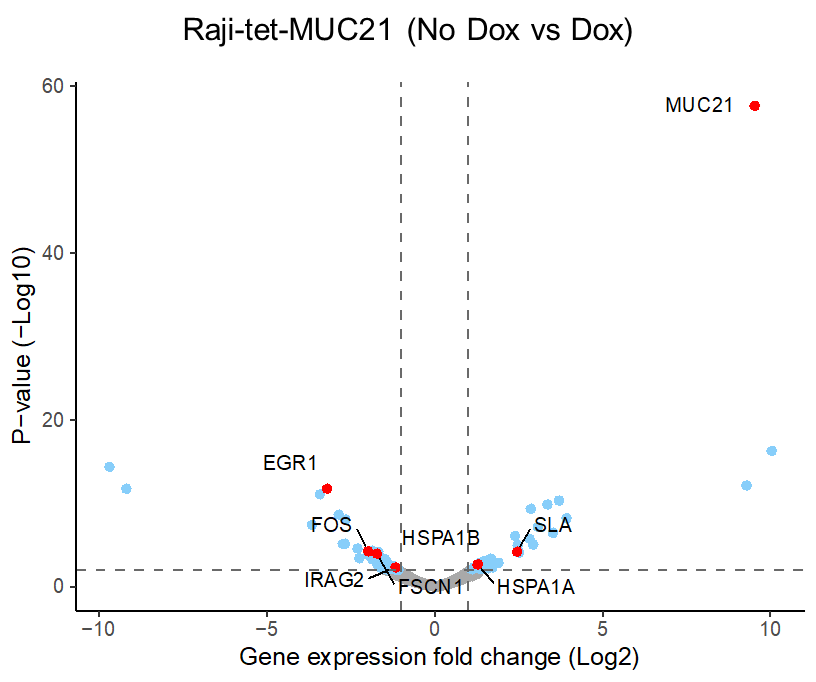
**

**Suppl. Figure S6.** A comparative transcriptome analysis was performed on Raji-tet-MUC21 cells using RNA sequencing to compare cells treated with and without Dox (1 μg/ml). The analysis involved identifying differentially expressed genes (DEGs) based on their p-value (shown by the horizontal dot line, below 0.01) and absolute log fold change (indicated by the vertical dot lines, above 1), calculated using edgeR software. The results are visualized in a volcano plot, where DEGs are represented by light blue and red dots, while gray dots indicate genes that did not exhibit significant differential expression between the two groups. Furthermore, genes associated with the "immune system" pathway in REACTOME are denoted by red dots, along with their respective gene names.

**
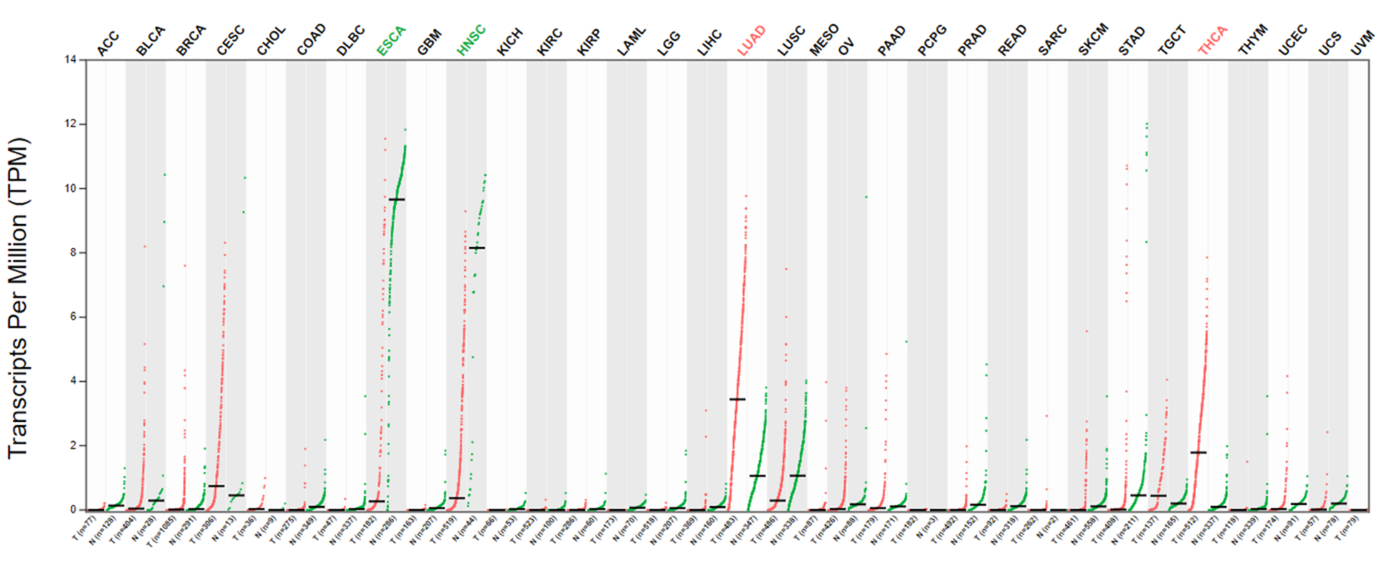
**

**Suppl. Figure S7.** A pan-cancer expression analysis of the *MUC21* gene using the GEPIA2 website, based on data from The Cancer Genome Atlas (TCGA). Tumor tissues (represented by red dots) correspond to TCGA tumors, while normal tissues (represented by green dots) include TCGA and GTEx normal tissues. Expression values are presented as log-normalized transcripts per million (TPM), and the median values are indicated by a horizontal black bar. The cancer type abbreviations are color-coded: red denotes a significant increase in *MUC21* gene expression in tumor tissues compared to normal tissues, while green denotes a significant decrease. TCGA abbreviations: ACC, adrenocortical carcinoma; BLCA, bladder urothelial carcinoma; BRCA, breast invasive carcinoma; CESC, cervical squamous cell carcinoma and endocervical adenocarcinoma; CHOL, cholangiocarcinoma; COAD, colon adenocarcinoma; DLBC, diffuse large B-cell lymphoma; ESCA, esophageal carcinoma; GBM, glioblastoma multiforme; HNSC, head and neck squamous cell carcinoma; KICH, kidney chromophobe; KIRC, kidney renal clear cell carcinoma; KIRP, kidney renal papillary cell carcinoma; LAML, acute myeloid leukemia; LGG, lower grade glioma; LIHC, liver hepatocellular carcinoma; LUAD, lung adenocarcinoma; LUSC, lung squamous cell carcinoma; MESO, mesothelioma; OV, ovarian serous cystadenocarcinoma; PAAD, pancreatic adenocarcinoma; PCPG, pheochromocytoma and paraganglioma; PRAD, prostate adenocarcinoma; READ, rectum adenocarcinoma; SARC, sarcoma; SKCM, skin cutaneous melanoma; STAD, stomach adenocarcinoma; TGCT, testicular germ cell tumors; THCA, thyroid carcinoma; THYM, thymoma; UCEC, uterine corpus endometrial carcinoma; UCS, uterine carcinosarcoma; UVM, uveal melanoma.

**
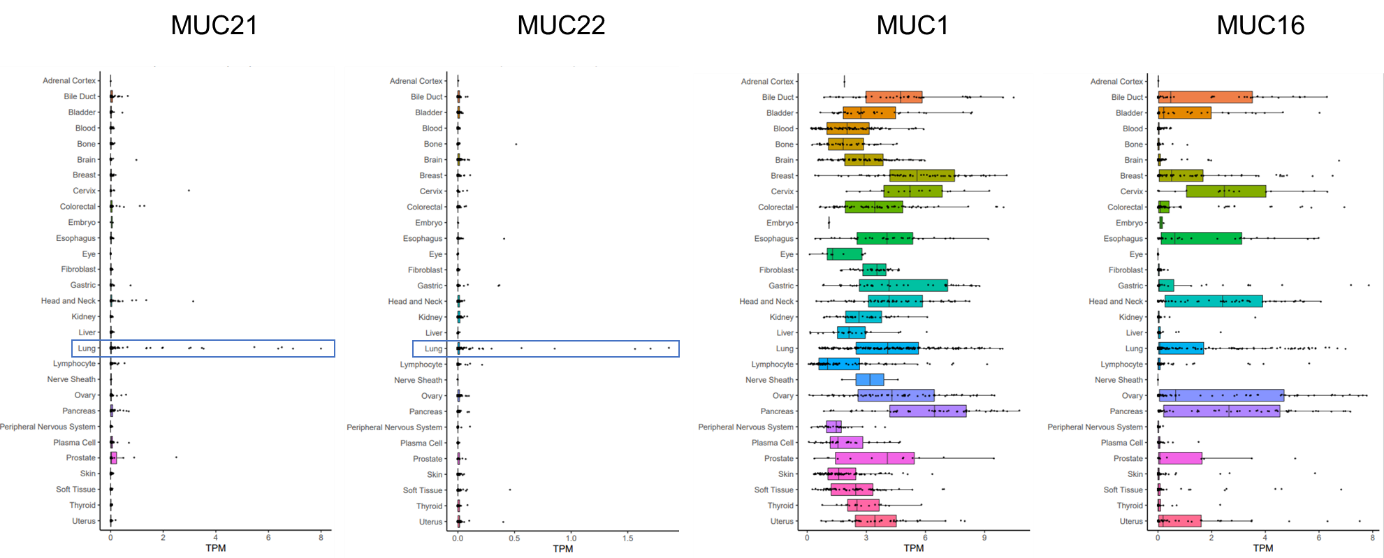
**

**Suppl. Figure S8.** The expression data for the *MUC1*, *MUC16*, *MUC21*, and *MUC22* genes in pan-cancer cell lines were extracted from the DepMap Public 21Q1 dataset available on the DepMap website. The analysis aimed to evaluate the expression levels of these genes from the mucin family, with a particular focus on the tissue of origin of the cancer cell lines. To visualize the data, box-and-whisker plots were generated using R software, representing the log-normalized TPM (transcripts per million) values. Each cell line was represented as a dot in the plots, and the median value was depicted by a black vertical line.


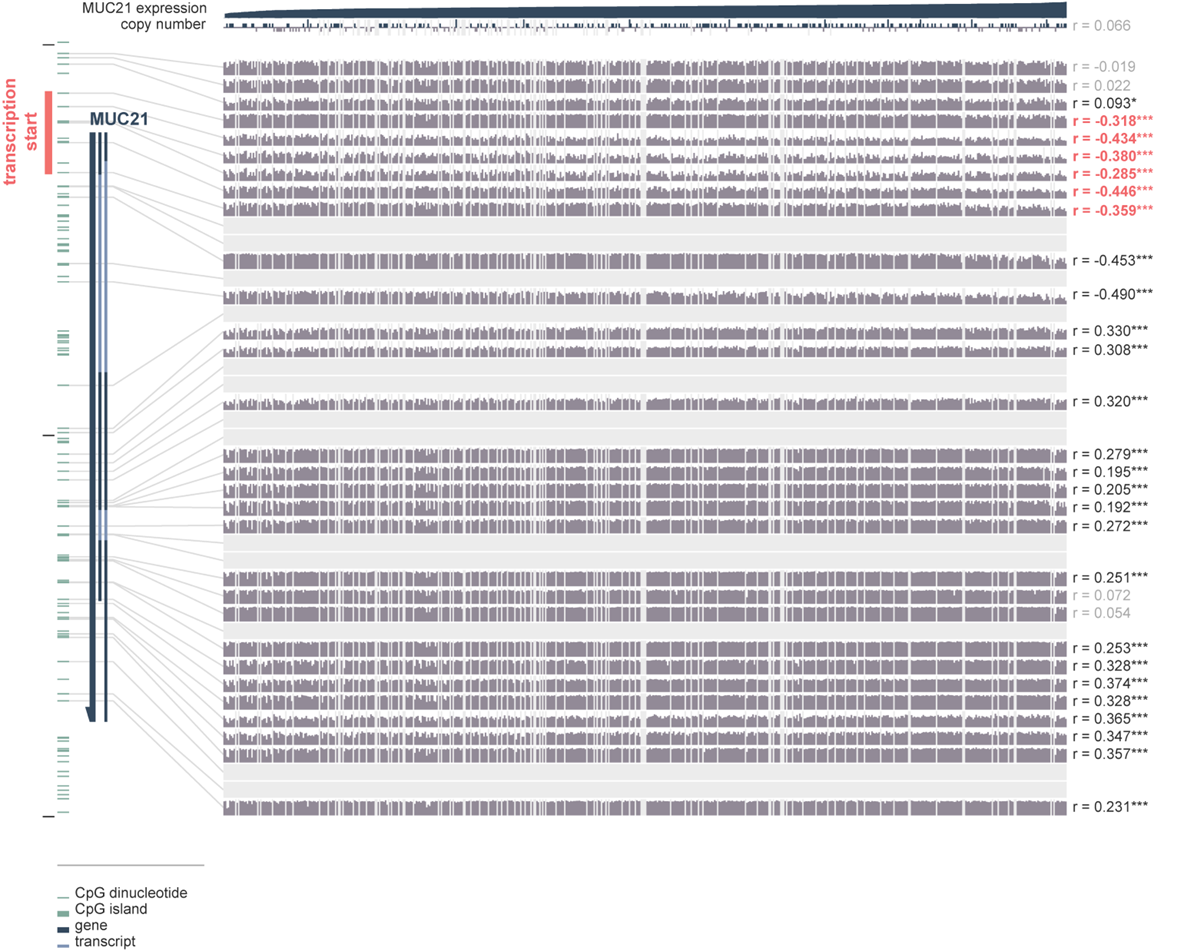


**Suppl. Figure S9.** The DNA methylation profile of the *MUC21* gene in TCGA LUAD dataset was analyzed using the MEXPERSS website. Each column and row in the analysis represents an individual sample and a specific genome location of the CpG site, respectively. On top of the analysis, the gene expression levels and DNA copy number variation of each sample were annotated. The correlations between gene expression and DNA methylation were indicated on the right side, using Pearson correlation coefficients and corresponding *P*-values (*< 0.05, **< 0.01, ***< 0.001, and ****< 0.0001).

**
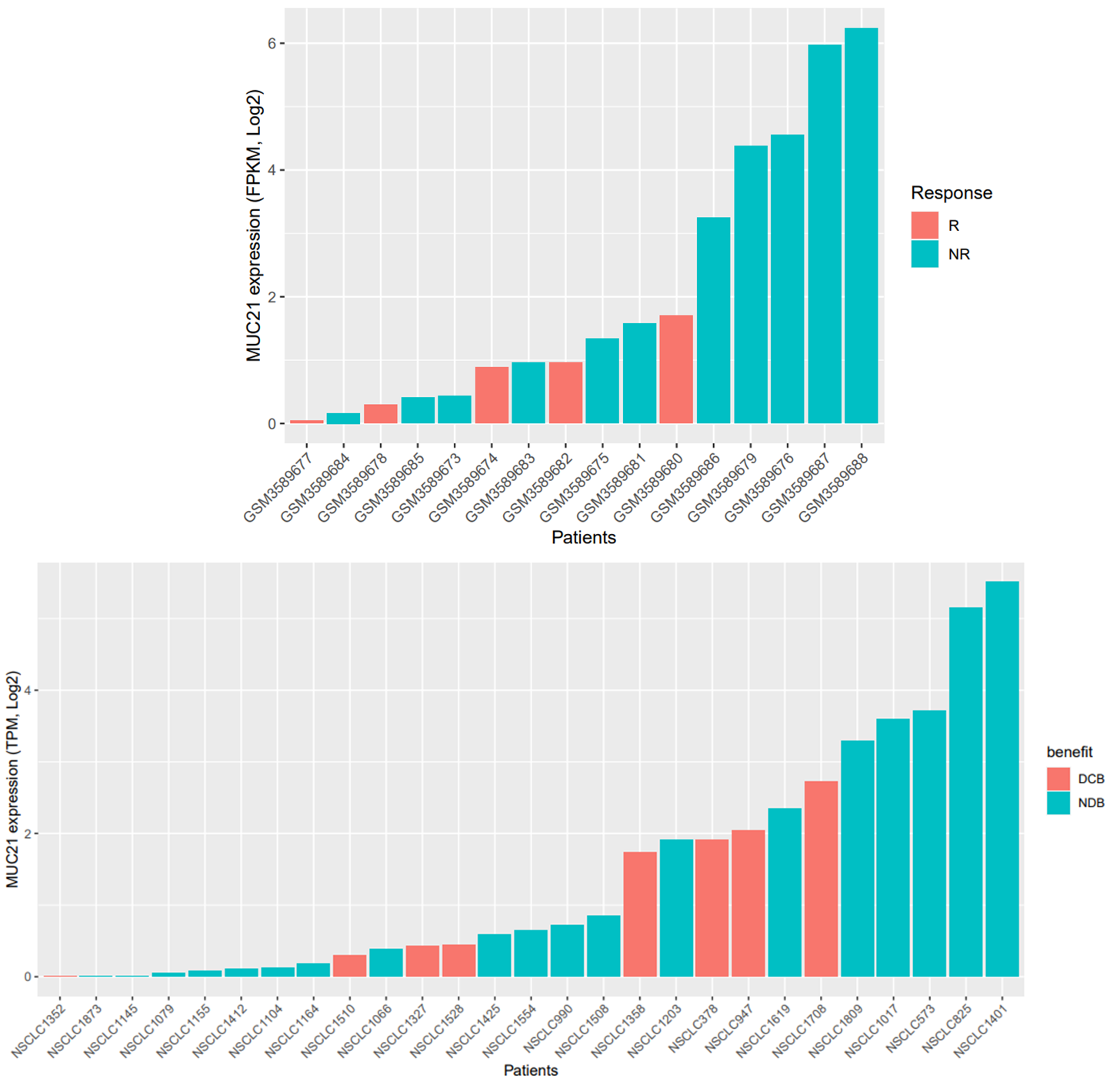
**

**Suppl. Figure S10.** The bar graph represents the MUC21 gene expression level of each individual (presented in Fig. 6F). The corresponding labels are as follows: R (responder), NR (non-responder), DCB (durable clinical benefit), and NDB (non-durable benefit).
